# The prospect of universal coronavirus immunity: characterization of reciprocal and non-reciprocal T cell responses against SARS-CoV2 and common human coronaviruses

**DOI:** 10.3389/fimmu.2023.1212203

**Published:** 2023-10-13

**Authors:** Mithil K. Soni, Edoardo Migliori, Jianing Fu, Amer Assal, Hei Ton Chan, Jian Pan, Prabesh Khatiwada, Rodica Ciubotariu, Michael S. May, Marcus R. Pereira, Valeria De Giorgi, Megan Sykes, Markus Y. Mapara, Pawel J. Muranski

**Affiliations:** ^1^ Columbia Center for Translational Immunology, Department of Medicine, Columbia University, New York, NY, United States; ^2^ Department of Medicine, Blood and Marrow Transplantation and Cell Therapy Program, Columbia University Irving Medical Center, New York, NY, United States; ^3^ Columbia University Medical Center, Herbert Irving Comprehensive Cancer Center, New York, NY, United States; ^4^ Department of Medicine, Division of Infectious Disease, Columbia University College of Physicians and Surgeons, New York, NY, United States; ^5^ Department of Transfusion Medicine, National Institutes of Health Clinical Center, Bethesda, MD, United States

**Keywords:** SARS-CoV-2, human coronavirus, *omicron*, multicoronavirus-specific T cells, cross-reactive T cells, SARS-CoV-2 variants

## Abstract

T cell immunity plays a central role in clinical outcomes of Coronavirus Infectious Disease 2019 (COVID-19) and T cell-focused vaccination or cellular immunotherapy might provide enhanced protection for some immunocompromised patients. Pre-existing T cell memory recognizing SARS-CoV-2 antigens antedating COVID-19 infection or vaccination, may have developed as an imprint of prior infections with endemic non-SARS human coronaviruses (hCoVs) OC43, HKU1, 229E, NL63, pathogens of “common cold”. In turn, SARS-CoV-2-primed T cells may recognize emerging variants or other hCoV viruses and modulate the course of subsequent hCoV infections. Cross-immunity between hCoVs and SARS-CoV-2 has not been well characterized. Here, we systematically investigated T cell responses against the immunodominant SARS-CoV-2 spike, nucleocapsid and membrane proteins and corresponding antigens from α- and β-hCoVs among vaccinated, convalescent, and unexposed subjects. Broad T cell immunity against all tested SARS-CoV-2 antigens emerged in COVID-19 survivors. In convalescent and in vaccinated individuals, SARS-CoV-2 spike-specific T cells reliably recognized most SARS-CoV-2 variants, however cross-reactivity against the *omicron* variant was reduced by approximately 47%. Responses against spike, nucleocapsid and membrane antigens from endemic hCoVs were significantly more extensive in COVID-19 survivors than in unexposed subjects and displayed cross-reactivity between α- and β-hCoVs. In some, non-SARS hCoV-specific T cells demonstrated a prominent non-reciprocal cross-reactivity with SARS-CoV-2 antigens, whereas a distinct anti-SARS-CoV-2 immunological repertoire emerged post-COVID-19, with relatively limited cross-recognition of non-SARS hCoVs. Based on this cross-reactivity pattern, we established a strategy for *in-vitro* expansion of universal anti-hCoV T cells for adoptive immunotherapy. Overall, these results have implications for the future design of universal vaccines and cell-based immune therapies against SARS- and non-SARS-CoVs.

## Introduction

Coronavirus Infectious Disease 2019 (COVID-19) caused by Severe Acute Respiratory Syndrome Coronavirus 2 (SARS-CoV-2), has resulted in over 6.5 million deaths worldwide (1). SARS-CoV-2 represents the third occurrence of a novel β coronavirus (CoV)-related disease in the last two decades. In 2003, SARS-CoV1 was identified in Asia, followed by Middle Eastern Respiratory Syndrome (MERS) CoV infection in 2012 (2, 3). These diseases had mortality rates of 9% and 40% respectively but have been confined to limited outbreaks (3–6).

Four endemic non-SARS human CoVs (hCoVs) circulate widely in the general population, including α-CoVs (hCoV-229E and NL63) and β-CoVs (hCoV-OC43 and HKU1) (7, 8). Endemic hCoVs cause up to 41% of seasonal upper respiratory infections (9, 10). While respiratory illnesses caused by endemic hCoV are typically self-limited, hCoV infection can be severe and protracted in patients with co-morbidities such as stem cell transplant (SCT) and solid organ transplant (SOT) recipients, leading to hospitalizations, oxygen use, intensive care admissions and even death (11). Thus, understanding endemic hCoVs infections is important, particularly for immunocompromised patients.

The role of cellular responses against SARS-CoV-2 and other hCoVs is not fully understood (12), although emerging data suggest that robust T cell immunity correlates with rapid resolution of COVID-19 (13–16). Competent adaptive cellular immunity alone may be sufficient for eradication of SARS-CoV-2 and recovery from COVID-19 in subjects with profound acquired or inborn defects in B cell function (17). Patients with suppressed humoral immunity due to leukemia or lymphoma can mount an effective immune response to SARS-CoV-2 if the T cell compartment is preserved (18). Moreover, while vaccine or infection-induced neutralizing antibody titers have limited half-life, anti-CoV T cell memory might be long-lived (19), as documented in survivors of SARS-CoV1 infection who retained T cell reactivity over 10 years after recovery (20, 21).

Emerging data suggest that previously acquired heterologous memory responses might explain the very broad spectrum of COVID-19 manifestations and disease severity among infected subjects, as previous “common cold” induced immunity likely conveys at least partial protection (22, 23). Unexposed individuals sometimes display SARS-CoV-2-specific T cell reactivity, conceivably induced by previous exposures to hCoVs that share some common epitopes (13, 24). Recent work by Kundu et al. indicates that pre-existing T cell responses may correlate with resolution of COVID-19 without seroconversion (25). However, the knowledge of T cell responses against the non-SARS hCoVs antigens remains limited, especially beyond the reactivity against S1 subunit and S2 subunit of spike protein targeted by vaccines. T cell reactivity against M and NP antigens from non-SARS hCoVs has not been fully explored.

Here we performed an in-depth analysis of CoV-specific T cell responses in healthy volunteers, (mostly healthcare workers, HCWs) with and without documented COVID-19 exposure as well as a cohort of high-risk immunocompromised patients (IP) including subjects with history of hematological malignancies, autologous and allogeneic SCT, SOT recipients and immunosuppressed patients with autoimmune diseases. T cell reactivity against the immunodominant antigens spike 1 and 2(S1, S2), membrane (M) and nucleocapsid (NP) proteins from SARS-CoV-2 was characterized in relation to responses against counterpart antigens from hCoV-229E, NL63, OC43 and HKU1. We also investigated cross-recognition of T cell responses against disparate epitopes derived from the spike antigen of multiple SARS-CoV-2 variants detected during the pandemic (26), including the highly mutated *omicron* variant (27, 28). Based on the observed cross-reactivity patterns, we postulate that a previously acquired infection or vaccination may provide broad T cell memory capable of recognizing, at least partially, future variants as well as related hCoV viruses. Finally, we established a strategy for *ex vivo* generation of universal multi-hCoV-specific T cells with enhanced ability to target common and emerging CoV (29). We postulate that this T cell product may be useful in the clinic as adoptive transfer therapy or prophylaxis for CoV infections in SCT recipients and other immunocompromised patients with impaired T cell immunity.

## Methods

### Patient blood sample collection and preparation

Healthy volunteers, primarily healthcare workers and associated individuals, with or without COVID-19 exposure, as well as immunocompromised patients were included after informed consent under an IRB approved protocol. Samples were collected serially during the early stages of the pandemic. COVID-19 exposure and vaccination status was documented at each collection point. Subjects with known history of positive COVID-19 test or documented positive COVID-19 serological test in the medical record were classified as exposed (COVID+). Subjects with no documented infection by COVID-19 PCR test and with recent negative results for serological testing were classified as “unexposed” (COVID-). Venous blood was collected for serum into a vacutainer containing no anticoagulant. Serum samples were obtained after clotting by centrifuging 3ml of whole blood at 3000rpm for 15 minutes. Serum aliquots were then stored in -80°C freezer. PBMCs were isolated using Lymphoprep™ density gradient medium (STEMCELL Technologies Inc., Canada) for the isolation of mononuclear cells, following the product’s protocol. Briefly, blood was diluted 1:1 with sterile dPBS, layered on Lymphoprep™ (ratio 1:1), and centrifuged 30 minutes at 1600rpm, without acceleration/brake. The PBMCs layer was carefully removed, and cells were washed twice with cytokine-free medium. PBMCs were counted, and immediately used for cell culture and/or flow cytometry analysis, or frozen using cryopreservation medium with 10% DMSO CryoStor® CS10 (STEMCELL Technologies Inc., Canada) and stored in liquid nitrogen.

### Generation of virus–specific T cells

Virus-reactive T cells were generated using commercially available overlapping peptide libraries against immunodominant viral antigens (S, M and NP), purchased from commercial vendors. Lits of all the peptide pools used in the study are listed in **Supplementary Table 2** along with their suppliers and catalog numbers. hCoVs (HKU1, OC43, NL63, 229E), or common viruses (ADV, BKV, CMV, EBV), were obtained from JPT Peptide Technologies or Miltenyi Biotec. SARS−CoV2 PepTivator® Peptide Pools, including the spike protein (PepTivator® SARS-CoV-2 Prot_S1, Prot_S2, Prot_RBD), the nucleocapsid phosphoprotein (PepTivator® SARS-CoV-2 Prot_N), and the membrane glycoprotein (PepTivator® SARS-CoV-2 Prot_M) were obtained from Miltenyi Biotec. The PepTivator® Peptide Pools are constituted by peptides of 15 amino acid length with 11 amino acid overlap. The peptides were grouped into different pools including pool S (equal amounts of Prot_S1, Prot_S2), pool NP, pool M. M and NP peptide libraries for hCoVs 229E, OC43, NL63 and HKU1 were custom synthesized at 70% purity (Peptides and Elephants; Germany). Cryopreserved PBMC were thawed and pulsed with peptide libraries (final concentration of 1μg/ml). After incubation, cells were suspended in CFM (Cytokine Free Media) media with interleukin-7 (IL-7; 10 ng/ml; Peprotech, NJ), and plated on 96 well U-bottom plates. IL-2 (30 IU/ml) was added after 72h. Cells were maintained and split as needed, every 3 days for approximately 14 days.

For the generation of clinical-grade (GREX®) T cell products, a comparable protocol has been used. PBMCs were pulsed with a master mix of other hCoVs spike S1 and S2 peptide pools, hCoVs membrane and nucleocapsid peptide pools, or SARS-CoV-2 spike S1 and S2, membrane and nucleocapsid peptide pools. After incubation, cells were plated in 6 well GREX® (Gas Permeable Rapid Expansion) plates, from Wilson Wolf (Saint Paul, MN), in CFM media with IL-7 and IL-15 (10 ng/ml; Peprotech, NJ). IL-2 (30 IU/ml) was added after 72h. Cells were maintained, fed, and split as needed, every 3 days for approximately 14 days. On day 14, cells were harvested and evaluated for antigen-specificity and functionality.

### Flow cytometry

All antibodies were procured from Biolegend (**Supplementary Table 3**; San Diego, CA) except for Viability Dyes (Miltenyi Biotec, FL). Flow cytometry was performed on PBMCs or cultured cells. Data was acquired on a BD Fortessa, and analysis was performed on FACS Diva and FlowJo software (BD Biosciences Corp, San Jose, CA, USA). PBMCs were resuspended in 1xPBS with live dead stain (1:200 dilution, Viability Dye, Miltenyi Biotec) for 10 minutes at room temperature. Samples were then resuspended in surface master mix and incubated for 20 minutes at 4°C. Cells were then washed twice in FACS buffer, fixed and permeabilized for intracellular analysis or resuspended in FACS buffer and acquired at the cytometer.

### Intracellular cytokine staining assay

For intracellular flow cytometry of T-cell cultures, cells were stimulated with viral peptide pools to a concentration of 1 mg/ml. 1 mg/ml of brefeldin A (Golgi PLUG, BD Biosciences) and 1 mg/ml of Monensin (Golgi STOP, BD Biosciences), anti-CD28 1µl/ml and anti-CD49d 1µl/ml were added to each well, and plates were incubated for 5h at 37°C 5% CO2. Cells were stained for surface markers following the previously described protocol (30). Intracellular cytokine staining was performed per manufacturer’s instructions using Fixation/Permeabilization Solution Kit (BD Bioscience), resuspended in FACS buffer and analyzed by flow cytometry.

### Activation-induced marker assay

Cells were cultured for 24 hours in the presence of indicated antigen pools in 96-wells U bottom plates at 0.5x10^6^ PBMC per well. Cells were stained with AIM markers CD137 and CD134. Stimulation with anti-CD3 antibody (OKT3) was included as a positive control.

### TCRβ CDR3 DNA sequencing

Virus-specific T cells were first expanded for 14 days as mentioned above. On day 14, the expanded cells were stimulated by cognate antigens in the presence of anti-TNF-α PE antibody and TNF-α Processing Inhibitor (TAPI) for 4 hours. Cells were then stained for CD3, CD4 and CD8 surface markers. Antigen-specific T cells defined as TNF-α^+^ were sorted using BD Influx cell sorter at the Columbia Center for Translational Immunology (CCTI) Flow Core. DNA from sorted cells was isolated using Qiagen’s DNA extraction kit (Cat#69504). Purified DNA was measured using nanodrop and sent to Adaptive biotechnologies for TCRβ CDR3 DNA sequencing.

### TCRβ CDR3 sequencing data processing and analysis

DNA was frozen down at -20C and shipped on dry ice to Adaptive Biotechnologies (Seattle, WA) for high throughput TCRβ sequencing. The TCR sequencing data were retrieved from Adaptive’s ImmunoSEQ software. PCR amplification, read sequencing, and mapping, with bias correction and internal controls, were performed by Adaptive Biotechnologies, returning tabulated template counts corresponding to unique bio-identity (CDR3 amino acid sequence + TRBV gene + TRBJ gene) across all samples. Analysis of TCRβ repertoire bulk DNA-seq data was performed in R, Rstudio and Microsoft Excel.

Clonality, which ranges from 0 to 1, is primarily used as a measure of diversity, such that higher clonality indicates less diversity (31). R20 is defined as the fraction of unique clones, in descending order of frequency, that cumulatively account for 20% of the sequenced repertoire: the higher the R20, the less immunodominance there is in a population. A standard quantitative measure of repertoire overlap is Jensen-Shannon divergence (JSD) (2), a tool that accounts for both clone number and frequency and is normalized on a scale of 0 to 1: a JSD of 1 indicates that all clones in 2 populations are distinct; a JSD of 0 indicates that all clones in 2 populations are identical. The code used to analyze TCRb bulk DNA-seq data and calculate clonality, R20 and JSD is available in previously published paper and has been deposited at https://codeocean.com/capsule/1539294/tree/v2. Clonal overlap of unique sequences among multiple targeted groups was shown in Venn diagrams, which was generated by an online software InteractiVenn (http://www.interactivenn.net) (32). Cumulative frequency was calculated as a percentage of all sequences weighted by copy numbers in designated populations (33). Top dominant sequences were ranked by their cumulative frequency within a designated sample.

COVID19-specific hits across genome figure were generated by Adaptive Biotechnologies’ immunoSEQ T-MAP COVID program (34, 35). Additional statistics and figures were generated using GraphPad Prism (GraphPad Software, La Jolla, CA).

### Data and materials availability

Raw TCRβ bulk DNA-seq data are freely accessible through https://clients.adaptivebiotech.com. The code used to analyze TCRβ bulk DNA-seq data and calculate clonality, R20 and JSD is available in our previously published paper [*Software Impacts*. 2021 (10) 100142] and has been deposited at https://codeocean.com/capsule/1539294/tree/v2.

### Quantification and statistical analysis

Graphs were produced, and statistical analyses were performed, using GraphPad Prism version 8.0 (GraphPad Software, Inc., San Diego, CA, USA). Simple linear regression was used to investigate T cell responses of individual antigens. To test the difference in paired observations Wilcoxon matched pairs signed rank test was used, while to compare ranks of unpaired observation Mann-Whitney test was used. P<0.05 is statistically significant.

Frequency of antigen specific T cells have been analyzed by either background subtracted data or stimulation index. Background subtracted data were obtained by subtracting the percentage of TNF-α^+^ cells after DMSO stimulation from the percentage of TNF-α^+^ cells after antigenic stimulation. Stimulation Index was calculated by dividing the percentage of TNF-α^+^ cells after SARS-CoV-2 stimulation with the percentage of TNF-α^+^ cells derived from DMSO stimulation (13).

## Results

### Microscale *ex vivo* priming and expansion strategy allows for sensitive gauging of virus-specific T cell immunity

**Figure 1:**
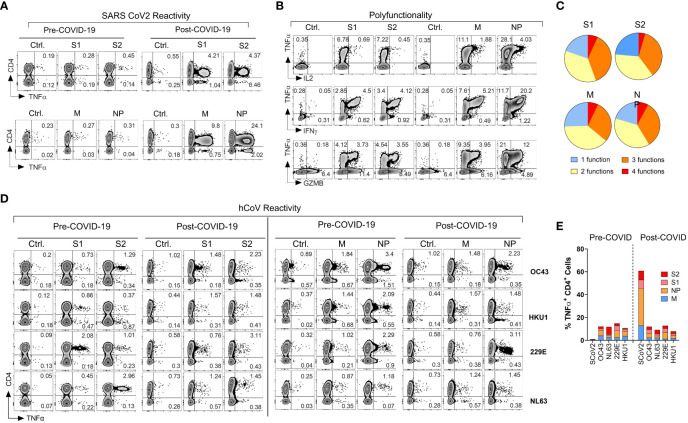
Emergence of potent polyfunctional T cell response following resolution of COVID-19 infection in a healthy subject. **(A)** Flow cytometric analysis of T cell populations generated upon *ex vivo* expansion of PBMCs from a healthcare worker (HCW) # 1008 using overlapping peptide libraries derived from indicated SARS-CoV-2 antigens. PBMCs were collected before and after documented COVID-19 infection. Zebra plots were gated first on viable CD3^+^ T cells followed by gating on both CD4^+^ T cells and CD8^+^ T cells. Therefore, CD4^-^ T cells in these plots represent CD8^+^ T cell response. The plots show intracellular production of TNF-α against indicated SARS-CoV-2 antigens at the end of 14-day *ex vivo* expansion. **(B, C)** Polyfunctionality analysis of SARS-CoV-2 reactive CD4^+^ T cells expanded *ex vivo* from post COVID-19 samples of the same donor. Zebra plots were gated on CD3^+^ CD4^+^ viable cells and show intracellular production of TNF-α vs. IFN-γ, vs. GZMB and IL-2 for each indicated SARS-CoV-2 antigen. Pie charts show the number of functions (single or multiple types of cytokine production) detected among antigen-reactive cells for each indicated viral antigen. **(D)** T cell reactivity in the HCW PBMC samples collected before and after COVID-19 infection expanded upon priming with S1, S2, M, or NP peptide mixes derived from hCoV (OC43, HKU1, 229E or NL63). **(E)** Frequencies of SARS-CoV-2 specific TNF-α^+^ cells among CD4^+^ T cells against indicated peptide mixes as compared to pre- and post-infection reactivity against indicated hCoVs.

Identification of T cell reactivity in peripheral blood mononuclear cells (PBMCs) by detection of cytokines *via* ELISPOT or flow cytometry has limited sensitivity due to the low frequency of antigen-specific memory cells in unmanipulated steady-state peripheral blood. This can be partially overcome using a sophisticated approach detecting activation-induced markers (AIM) that are upregulated upon stimulation, allowing for sensitive identification of antigen-specific CD4^+^ T helper (Th) cells (36, 37). However, a large number (0.1-2x10^7^) of PBMCs (24, 38) are required to detect a meaningful signal above the background. Based on our previous experience, we hypothesized that a microscale priming/expansion strategy might unequivocally detect pre-established T cell immune responses against viral antigens even when the frequency of the memory T cells is at the background level (39). Using overlapping peptide libraries (pepmixes) composed of 15-mer peptides covering full-length immunodominant viral antigens of interest, we directly compared reactivity within PBMC samples (n=8) upon baseline stimulation (Day 0) and following a 14-day *in vitro* expansion. We assessed the baseline reactivity of PBMCs using AIM assay (**Figure S1A** upper panel), which detects upregulation of CD134 and CD137 (36), and the intracellular cytokine secretion (ICS) assay, which detects secretion of TNF-α and IFN-γ (**Figure S1A** middle panel). The *ex vivo* expanded samples from the same donors were tested by ICS assay upon antigenic re-challenge on the day 14 from the start of culture (**Figure 1A**, bottom panel). The gating strategy to measure antigen specific T cells by AIM assay or ICS assay is illustrated in **Supplementary Figures S1B**, **S1C** respectively. The set of peptide libraries included common viral antigens EBV, BZLF and EBNA1, Adenovirus (AdV) penton (Ad5), BK virus large T (LT) and VP1 antigens as well as the S1, S2, M and NP antigens from SARS-CoV-2, HKU1 and 229E hCoVs. In some donors the antigen-specific cytokine production was detectable upon direct stimulation (**Figure S1D**), while the AIM method revealed reactivity in a larger portion of tested subjects (**Figure S1E**). However, significantly more reactive donors were identified upon 14-day *in vitro* priming/expansion, indicating improved sensitivity of the proposed strategy (**Figures S1C, S1E**). Importantly, this approach unmasked otherwise missed reactivity against viral antigens from both tested hCoVs. Thus, direct detection of reactivity on Day 0 (Baseline) by ICS or AIM (to a lesser degree) was affected by low dynamic range and background signal, whereas the magnitude of the responses in the expanded T cell populations was clearly above background (**Figure S1A**), enabling unequivocal identification of reactive T cells. Overall, the micro-scale priming/expansion strategy represents a reliable tool for gauging the functional immunocompetence and immune reactivity against the viral antigens of interest, especially when a low frequency of precursors is present and a limited quantity of starting PBMCs is available for analysis.

### Induction of a broad and robust antigen-specific T cell reactivity against SARS-CoV-2 in a healthy donor following the resolution of COVID-19

During the early stages of the pandemic, we collected serial PBMC samples from healthy volunteers (healthcare workers) and immunocompromised subjects with or without documented COVID-19 exposure to investigate T cell immune responses against SARS-CoV-2 and related 229E, OC43, NL63 and HKU1 hCoVs. One of the subjects, #1008, a healthcare worker involved in direct patient care, developed PCR-documented COVID-19 approximately three months after initial sample collection, thus allowing for investigation of T cell responses pre- and post- SARS-CoV-2 infection. Pre-COVID T cell responses against SARS-CoV-2 S1, S2, M and NP antigens were minimally detectable above background (**Figure 1A**), confirming naïve/unexposed status. In post-COVID-19 samples collected 14 days after the day of documented infection, a marked increase in reactivity against all four tested SARS-CoV-2 antigens was observed, predominantly within the CD4^+^ T cell compartment. The maximal reactivity was seen against NP antigen with 25.52% TNF-α^+^ cell among CD4^+^ T cells, followed by M (10.29%), S2 (6.14%) and S1 (5.86%; **Figure 1A**). Furthermore, the reactive CD4^+^ T cells displayed a significant polyfunctionality with antigen-specific secretion of TNF-α, IFN-γ, granzyme B (GZMB) and IL-2 with 6.85% of S1 reactive cells, 4.55% of S2 reactive cells, 7.07% of M reactive cells and 7.86% NP reactive cells displayed all 4 functions (**Figures 1B, C**). We then tested reactivity to the corresponding immunodominant antigens from the related common α- and β-hCoVs in the same donor. The magnitude and pattern of reactivity against all non-SARS hCoV targets pre- and post-COVID-19 remained low and was minimally affected by COVID-19 (**Figures 1D, E**). Thus, this HCW acquired robust and highly focused antigen-specific T cell memory against SARS-CoV-2 post-infection. Furthermore, SARS-CoV-2-specific T cell responses involved mainly CD4^+^ Th cells with minimal response in CD8^+^ compartment (**Figure 1A**, CD4^-^TNFα^+^ quadrant). However, *ex vivo* reactivity against antigens derived from the common viral pathogens CMV (pp65 and IE-1), EBV (EBNA1 and BZLF1), BK (LT and VP1) and AdV (Ad5) was seen in either CD4^+^ and/or CD8^+^ T cell compartments of the same subjects (**Figures S1F, S1G**). This suggests that the observed CD4 or CD8 reactivity patterns against each viral antigen depend on the pre-established *in vivo* memory inherent to each virus and is driven to a lesser degree by the fundamental tendency of the assay to detect only CD4^+^ T cell responses.

### T cell responses against SARS-CoV-2 antigens among healthcare workers and immunocompromised patients

**Figure 2:**
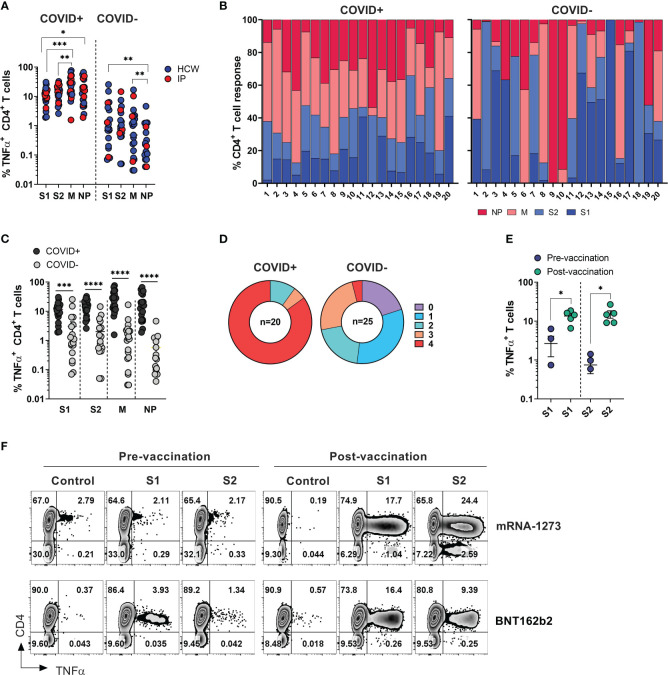
SARS-CoV-2 specific T cell responses among HCW and immunocompromised patients with and without history of COVID-19. PBMC samples were collected from healthy volunteers (who are healthcare workers) and immunocompromised (IP) subjects exposed or unexposed to SARS-CoV-2 and expanded *in vitro* for 14 days upon priming with SARS-CoV-2 peptide mixes S1, S2, M and NP. Reactivity (%TNF-α^+^ CD4^+^T cells) was evaluated in the final cultures upon re-stimulation with cognate peptide mixes. **(A)** Frequencies of TNF-α secreting cells among CD3^+^CD4^+^ T cells recognizing indicated SARS-CoV-2 peptide mixes in *ex vivo* expanded cultures. Magnitude of reactivity against peptide mixes in COVID+ and COVID- cohorts was compared using Wilcoxon matched-pairs signed rank test. **(B)** Relative contribution of all four SARS-CoV-2 antigens to total CD4^+^ T cell response against SARS-CoV-2 antigens in individual donors is shown in samples from COVID+ (n=20) and COVID- (n=25) donors. (5 COVID- donors did not show response to any of the 4 antigens.) **(C)** CD4^+^ T cell reactivity against indicated SARS-CoV-2 peptide mixes among COVID+ and COVID- donors. Statistically significant differences of reactivity between two groups were analyzed by Mann-Whitney test. **(D)** Percentage of COVID+ (n=20) and COVID- (n=25) donors recognizing (frequency of CD4^+^TNF-α^+^ cells above 0.5%) one or more SARS-CoV-2 antigens. **(E)** Frequencies of SARS-CoV-2 S1 and S2 reactive CD4^+^ T cells in otherwise unexposed HCWs pre- and post-vaccination. Statistically significant differences of reactivity were determined by Wilcoxon matched pairs signed rank test. Horizontal lines indicate the mean ± SEM. *P < 0.05, **P < 0.01, ***P < 0.001, **** p-value < 0.0001. **(F)** Representative zebra plots demonstrating antigen-specific T cell responses against S1 and S2 antigen pre- and post-vaccination.

Next, we conducted an analysis of *ex vivo* T cell responses targeting the immunodominant antigens of SARS-CoV-2 in a cohort comprising healthcare workers (HCW; n=32) and immunocompromised patients (IP; n=13; see **Supplementary Table 1**). Both unexposed (COVID-; n=25) and exposed (COVID+; n=20) individuals were included in the cohort. In line with the case presented in **Figure 1**, convalescent donors mounted robust *ex vivo* CD4^+^ T cell reactivity against SARS-CoV-2 S1, S2, NP, and M antigens (**Figure 2A**, left panel). CD8^+^ T cell responses were also seen in most of these donors, albeit with a somewhat more variable pattern (**Figure 2SA**).

Among COVID+ donors, the anti-M and NP responses were more prominent in comparison to the reactivity against S1 and S2 antigens (**Figures 2A, B**, left panels). Unlike COVID+ donors, COVID- subjects sampled during the early stages of the pandemic displayed a highly variable reactivity pattern dominated by immune responses targeting the S1 and S2 antigens (**Figures 2A, B**, right panels). These findings indicate that COVID+ individuals exhibited diverse and extensive reactivity against all tested antigens, with a relatively prominent contribution from non-spike NP and M-specific activity. Conversely, COVID- individuals with anti-SARS-CoV-2 reactivity exhibited narrower and more focused immune responses primarily directed towards Spike antigens.

As expected, the magnitude of *ex vivo* SARS-CoV-2 antigen responses, measured by frequency of TNF-α^+^ T cells, was significantly higher in COVID+ subjects compared to COVID- subjects within both the CD4^+^ and CD8^+^ T cell compartment (**Figures 2C**, **S2B**). All the COVID+ subjects included in the study exhibited a response to at least two antigens, with 90% of subjects showing reactivity against at least 3 out of the 4 tested SARS-CoV-2 antigens, and 85% of subjects robustly responding to all four antigens (**Figure 2D**, left panel). Among the COVID- and unvaccinated individuals, 5 participants (20%) did not display any reactivity (defined as 0.5% above background) against any of the tested SARS-CoV-2 antigens. Eight subjects (32%) exhibited a clear response to at least one antigen, while six subjects (24%) demonstrated reactivity against three antigens (**Figure 2D**, right panel). Notably, one subject (#1018), a healthcare worker who had been quarantined in March 2020 after a self-limiting non-febrile upper respiratory tract illness following close contact with a confirmed COVID-19 patient, consistently tested negative by PCR and serological studies but displayed a robust T cell response to all four SARS-CoV-2 antigens (**Figure S2C**, upper row). Similarly, the immunocompromised subject -exhibited a strong T cell response against three SARS-CoV-2 antigens (**Figure S2C**, lower panel). These data suggest that both subjects might have developed a mild infection without seroconversion, but with the emergence of vigorous T cell responses.

There was no statistically significant difference in the magnitude of CD4^+^ T cell responses against the tested SARS-CoV-2 antigens between COVID+ individuals who were healthcare workers (HCWs) and those who were immunocompromised (**Figure S2D**, left panel). However, there was a trend indicating more potent reactivity specific to M and NP antigens among healthy subjects (HCWs), although this analysis may be limited due to the small sample size of non-healthcare worker COVID-19 survivors. A similar trend was seen within the CD8^+^ T cell compartment; there was a trend towards lower responses in convalescent immunocompromised subjects (**Figure S2D**, right panel).

Finally, significant increase in the frequencies of T cells specific to the spike protein compared to pre-vaccination samples from the same individuals (**Figure 2E**). Representative dot plots illustrate robust T cell responses against both subunits of spike protein emerged in the donors who received either BNT162b2 or mRNA-1273 vaccines (**Figure 2F**). Thus, natural infection with SARS-CoV-2 or vaccination typically induces T-cell immunity against COVID-19.

### T cell recognition of the divergent epitopes from the emerging variants of SARS-CoV-2 is largely preserved

The emergence of SARS-CoV-2 variants, including the delta and *omicron* BA.4, BA.5 variants, has raised concerns regarding their ability to evade the immune response, a phenomenon that has been extensively documented for humoral immunity (27, 40). To examine whether previously acquired natural or vaccination-induced T cell immunity exhibits cross-reactivity against SARS-CoV-2 variants, PBMCs from COVID+ (n=6) or unexposed (n=6) subjects who received one of three approved SARS-CoV-2 vaccines were expanded *in vitro* using full-length ancestral S1/S2 pepmixes. The resulting T cells were then examined for reactivity against peptide pools of eight SARS-CoV-2 variants spanning mutated regions of S protein and compared it to the matching wild type (WT) antigen pools from the ancestral virus. Response to each variant pool was expressed as stimulation index (S.I), calculated by dividing the percentage of TNF-α^+^ cells after SARS-CoV-2 stimulation with the percentage of TNF-α^+^ cells derived from DMSO stimulation and then compared to WT-counterpart.

**Figure 3:**
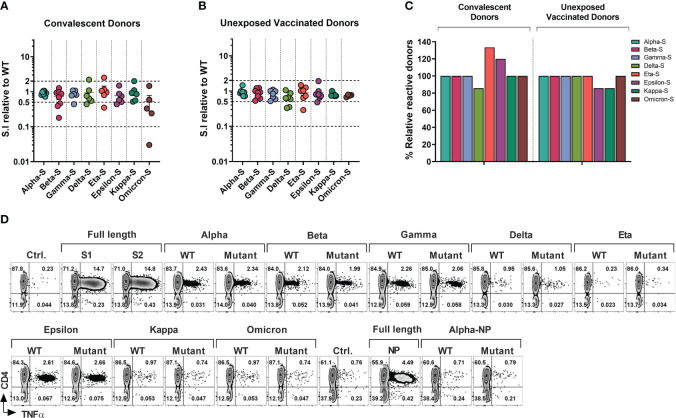
T-cell Response to SARS-CoV-2 Variants of Concerns and Variants of Interests in Healthcare Workers and Immunocompromised Patients. T cell cultures primed against SARS-CoV-2 Spike (S1/S2) and NP peptide libraries were generated from convalescent donors and unexposed fully vaccinated donors. **(A, B)** Relative response against variant antigen pools as compared to their WT counterpart expressed as stimulation index (S.I) in convalescent donors **(A)** and unexposed vaccinated donors **(B)**. **(C)** Proportion of donors displaying unequivocal reactivity against variants relative to WT counterpart of indicated SARS-CoV-2 variants among convalescent donors (left panel) and unexposed vaccinated donors (right panel). **(D)** Representative flow cytometric analysis illustrating antigen-specific intracellular TNF-α secretion upon stimulation with SARS-CoV-2 variants as compared to the counterpart WT peptide pool in S and NP cultures from a vaccinated HCW with no history of COVID-19.

Among COVID+ donors the overall SARS-CoV-2-specific CD4^+^ T cell response against all eight variants was largely preserved (S.I range 1.14 to 0.53; **Figure 3A**). Mean reductions of 27.87%, 22.5% and 16.17% were observed against *beta, epsilon* and *gamma* variants respectively, while only a marginal decline was seen against *alpha* (8.5%), *delta* (5.17%) and *kappa* (0.83%) variants. The greatest mean reduction of 47% was observed in the case of *omicron*, consistent with the high mutation burden of this variant. Interestingly, a higher mean CD4^+^ T cell response (S.I 1.14) was seen against the *eta* variant. At an individual level, a maximum of 33-fold reduction was observed in a single COVID+ IP subject against *omicron* variant, while two additional donors showed more than 2-fold reduction (**Figure 3A**). No donors showed more than a 10-fold reduction in the magnitude of CD4^+^ T cell response towards other variants. 3 donors showed more than 2-fold reduction (but less than 10-fold; S.I range 0.18-0.46) in reactivity against *beta*. Relatively robust cross-reactivity was seen against all the other variants tested. Notably, some COVID+ donors demonstrated more than 2-fold increase in response to the variants compared to the ancestral pool, namely one donor each against *delta*, *eta*, and *kappa* variant, possibly due to infections with these variants.

In the vaccinated COVID- group (**Figure 3B**), mean CD4^+^ T cell S.I was 0.9413 to 0.6414 relative to WT counterpart, with maximum mean reduction of 35.86% seen against *delta* variant followed by 25.75% mean reduction towards *omicron*. *Kappa, gamma*, and *beta* variants displayed mean reduction of 21.14%, 18.29% and 11.12% respectively. Less than 10% of mean reduction in CD4^+^ T cell response was observed in case of *alpha* (5.87%), *eta* (8.14%) and *epsilon* (9.14%) variants. At an individual level, only one donor failed to cross-react with *epsilon* and *kappa* pools, while retaining the reactivity towards the control WT peptide pools (**Figure 3B**). None of the other donors displayed more than 10-fold loss of response compared to WT peptide pool apart from the *epsilon* and *omicron* variants, with maximum of 3.44-fold reduction observed in a vaccinated HCW against eta variant. Only 2 other HCW donors showed more than 2-fold reduction against the *delta* variant (3-fold and 2.86-fold reduction).

In summary, all tested COVID+ survivors who exhibited reactivity towards the WT-peptide pool also demonstrated cross-recognition of all other variants, except for the *delta* variant, which had fewer individuals showing reactivity compared to the WT counterpart (5 out of 6 individuals, or 83.33%, for WT vs 4 out of 6 individuals, or 66.67%, for the *delta* variant). Curiously, fewer subjects recognized WT pools compared to the mutant pools of the *eta* or *epsilon* variants (4/6 vs 5/6 and 5/6 vs 6/6 respectively) (**Figure 3C**, left panel). In the COVID- subgroup, apart from one donor *epsilon* and *kappa* variant all the donors that displayed a response to WT pools also cross-recognized other variants (**Figure 3C**, right panel).

**Figure 3D** shows a representative T cell response of a COVID- vaccinated HCW donor against all tested variants sub-pool. Importantly the responses analyzed in above set of experiments involved detection of reactivity against the small number of variant epitopes spanning only divergent regions (**Figure S3A**) and therefore represent only a minor portion of the overall T cell immunity against the full-length Spike proteins, while among COVID-19 survivors the emerging T cell repertoire targets hundreds of epitopes (see **Figure 2**). Similarly, total anti-Spike T cell reactivity induced by vaccination (**Figure 3D**) is much more comprehensive. Thus, the broad T cell immunity against SARS-CoV-2 might provide substantial cross-protection against emerging variants, as it targets full length of the immunodominant viral proteins.

### Cross-reactivity is partially preserved between Spike-specific T cell responses against the ancestral and *omicron* variant

The recent dominant *omicron* variant harbors the highest number of amino acid alterations (37 mutations) within the Spike protein as compared to previous variants. Furthermore, *omicron* is more transmissible than previous variants. It can evade neutralizing antibody responses and has a greater capacity for reinfection (41–43). Recent studies have examined T cell responses to *omicron* BA.1 spike protein (44, 45).

However, the extent to which *omicron*-specific T cells can recognize the spike protein of the ancestral virus or other variants has not been thoroughly investigated. Consequently, we conducted a thorough investigation of T cell responses against spike protein of both omicron variant and the ancestral virus. To accomplish this, we utilized complete S1/S2 peptide libraries of the *omicron* variant and ancestral virus to prime peripheral blood mononuclear cells (PBMCs) obtained from individuals who had either been infected with COVID-19 or were unexposed to COVID-19 but were vaccinated. The resulting T cell populations were then examined to evaluate their ability to cross-recognize mutated epitopes within the spike protein of specified variants.

**Figure 4:**
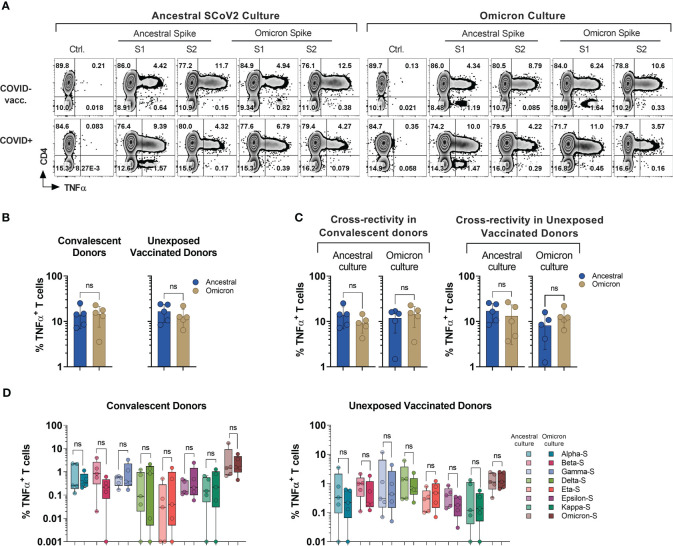
Cross-recognition of T cells specific to ancestral and *omicron* spike antigens. PBMCs from convalescent (n=5) and vaccinated but otherwise unexposed donors (n=5) were primed and expanded using full-length ancestral and *omicron* Spike peptide mixes. Reactivity was tested against the indicated antigens. **(A)** Representative flow cytometric analysis of antigen-specific reactivity (TNF-α secretion) in cultures from convalescent donor (#1030) and unexposed vaccinated (#1007) donors depicting T cell response to ancestral and *omicron* full length spike protein pool. **(B)** Frequency of antigen-specific T cells generated against ancestral and *omicron* S peptide mixes and tested against cognate antigens. **(C)** Frequency of cross-reactive T cells between ancestral and *omicron* Spike cultures, as compared to cognate peptide mix reactivity. **(D)** Comparison of cross-recognition of indicated SCoV2 variants in cultures initially generated by priming with full length ancestral or *omicron* spike peptide mixes. Statistically significant differences of reactivity were determined by Wilcoxon matched-pairs signed rank test. ns=p>0.0332.

Among all the donors tested (n=10; five COVID+ and five COVID- vaccinated), there was no statistically significant difference observed in the frequency of S-reactive T cells (TNF-α^+^) following ex vivo expansion with either *omicron* or ancestral S1/S2 antigens (**Figures 4A, B**). Although, the reactivity was reduced upon cross-stimulation as compared to stimulation with the cognate antigen, it did not reach statistical significance in either of the subgroups. (**Figure 4C**). The overall magnitude of the ancestral S-specific T cell response among COVID+ subgroup when challenged with *omicron* spike library showed a mean loss of 33.25%. This finding suggests that 66.75% of the reactivity against the *omicron* variant was preserved. Alternatively, when the *omicron* spike-specific T cell responses were subjected to the ancestral spike, there was an average decrease of 17.7% compared to the response elicited by the cognate antigen (**Figure 4C**, left panel). Similarly, among COVID- subgroup, Ancestral spike specific T cells retained app. 77.52% of the reactivity when challenged with *omicron* spike peptide library and *omicron* spike specific T cells challenged with ancestral spike peptide library, it showed mean reduction of 36.34% (**Figure 4C**, right panel). These results indicate that despite the mutations, robust cross-recognition can be observed between ancestral virus and *omicron* variant.

To investigate the cross-recognition capability of *omicron*-primed T cells in comparison to the ancestral spike, we conducted a challenge by exposing these T cells to antigens derived from variants of concern (VOCs) and variants of interest (VOIs) (**Figure 4D**). All the variant pools used in the study displayed comparable cross-recognition between *omicron* and ancestral S-specific T cell cultures (**Figure 4D**). These results suggest a possibility that natural infection or vaccination might provide protection against the variants tested in the study.

Additionally, subject #1008 (see **Figure 1**) who developed natural immunity during the early stage of pandemic (Ancestral for infection #1 and Omicron infection #2), received full course of vaccination and subsequently developed reinfection during the 4^th^ wave dominated by *omicron* variant (**Figure S3B**), allowing for unique longitudinal analysis of CD4^+^ and CD8^+^ T cell responses. Marked S-specific T cell reactivity was consistently seen following the initial infection, the course of vaccination, and re-infection with likely *omicron* variant, but the magnitude of anti-NP and M- T cells responses relatively declined as compared to the primary infection (**Figures S3C, S3D**).

We then compared anti-Spike reactivity of T cell cultures from early and late time points upon priming with full-length S1/S2 pepmixes of ancestral virus and *omicron* variant and tested their ability to cross-recognize other variants. The post-reinfection sample displayed enhanced CD4^+^ and CD8^+^ T cell responses against full-length *omicron* pool in contrast to the initial draw, suggesting variant-specific priming. As expected, *omicron* S-specific T cells mounted a more potent response to cognate *omicron* sub-pool, but they also produced a relatively higher response against the *kappa* variant. Other variants displayed a similar response between both ancestral and *omicron* S-specific T cells, indicating preserved cross-reactivity (**Figure S3E**). In summary, the magnitude of response against the *omicron* S1/S2 antigens is reduced by at least half following COVID-19 or vaccination with the ancestral variant. Nonetheless, prior vaccination or previous COVID-19 infection can induce cross-prime against *omicron*.

### T cell responses against common hCoV antigens are seen in COVID-naïve and COVID+ individuals

**Figure 5:**
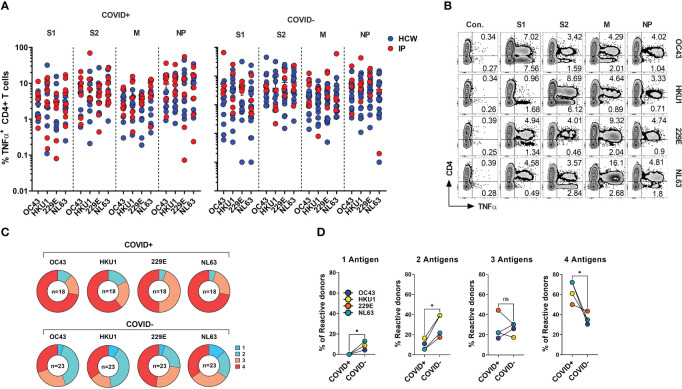
CD4^+^ T cell responses against the immunodominant antigens from “common cold” hCoVs in HCWs and IPs. T cell cultures from indicated donors were generated upon priming with peptide mixes for S1, S2, M and NP antigens of α-hCoVs 229E and NL63 and β-hCoVs OC43 and HKU1 and tested as day 14 for reactivity. **(A)** Frequencies of antigen-specific CD4^+^ T cells reactive (TNF-α^+^) to S1, S2, M and NP antigens in samples obtained from COVID+ (left panel) and COVID- (right panel) donors. **(B)** Representative example of flow cytometric analysis illustrating broad anti-hCoV response against S1, S2, M and NP peptide mixes in a subject with documented COVID-19 exposure (#1015). **(C)** Percentage of donor samples with reactivity against one or more antigens of all analyzed hCoVs among COVID+ and COVID- subgroups. Recognition was defined as frequency of antigen-specific TNF-α secreting T cells >0.5%. **(D)** Frequency of donors displaying reactivity against either one, two, three or four antigens (S1, S2, M, NP) of indicated hCoVs in COVID+ and COVID- cohorts. Statistically significant differences of reactivity was determined by Wilcoxon matched-pairs signed rank test. *P < 0.05. ns, not significant.

SARS-CoV-2 shares the general structure and at least partial sequence homology with the corresponding structural proteins of the endemic hCoVs (24, 46). Consequently, individuals with previously developed immune memory against these viruses may exhibit cross-recognition of COVID-19, potentially providing them with partial protection. Conversely, there might be a broader response to non-SARS hCoVs following the recovery from COVID-19. To better characterize the spectrum of anti-hCoV T cell responses we tested *ex vivo* reactivity using the custom set of pepmixes derived from S1, S2, M and NP antigens of all four endemic hCoVs. A spectrum of antigen-specific activity among COVID-19 survivors and unexposed COVID- subjects was observed, involving both Spike and non-Spike antigens (**Figure 5A**), likely representing an imprint of past community-acquired endemic hCoV infections, as illustrated in **Figure 5B** depicting recognition of all antigens from multiple hCoVs in a COVID+ donor. Both COVID+ and COVID- donors also displayed vigorous, but highly variable antigen-specific CD8^+^ T cell responses (**Figures S4A, S4B**). Overall, reactivity to at least one of the antigens of each hCoVs (**Figure 5C**) was found in all tested individuals. However, concurrent robust responses (%TNF-α^+^ >0.5%) against all four antigens from each hCoV were significantly more prevalent in COVID+ samples than COVID- samples (**Figure 5D**). To further examine whether previous SARS-CoV-2 infection affects reactivity towards hCoVs, we inspected the magnitude of responses against hCoVs in COVID+ and COVID- donors. A significantly higher reactivity was seen against S1 antigen of OC43 and NL63 among COVID-19 survivors as compared to COVID- subjects and against S2 antigens of HKU1 and NL63 with a trend towards increased responses against S2 OC43 and 229E, as well as a significantly higher reactivity against M and NP of NL63 among COVID+ subjects (**Figure S4C**). This was further underscored upon Pearson correlation analysis showing significant correlation between SARS-CoV-2 responses and corresponding responses directed against S2 (r^2^ = 0.5677) and M (r^2^ = 0.6757) of OC43; NP antigens of HKU1 (r^2^ = 0.5057) and OC43 (r^2^ = 0.6406) (**Figure S4D**). When responses against α-hCoVs were analyzed, significant correlation was seen between reactivity directed against S2 of SARS-CoV-2 and S2 of NL63 (r^2^ = 0.5842) and M antigen of 229E (r^2^ = 0.4357) (**Figure S4E**). Overall, these data suggest a potential link between T cell immunity emerging post-COVID-19 and reactivity potentially directed against other members of CoV family, suggesting possible cross-reactivity and perhaps cross-protection. Indeed, certain COVID-19 survivors mounted broad and robust anti-CoV T cell responses directed against the majority of antigens from all analyzed hCoVs.

### T cell responses against hCoV antigens display broad cross-reactivity, but responses against SARS-CoV-2 in COVID-19 survivors are distinct and specific

**Figure 6:**
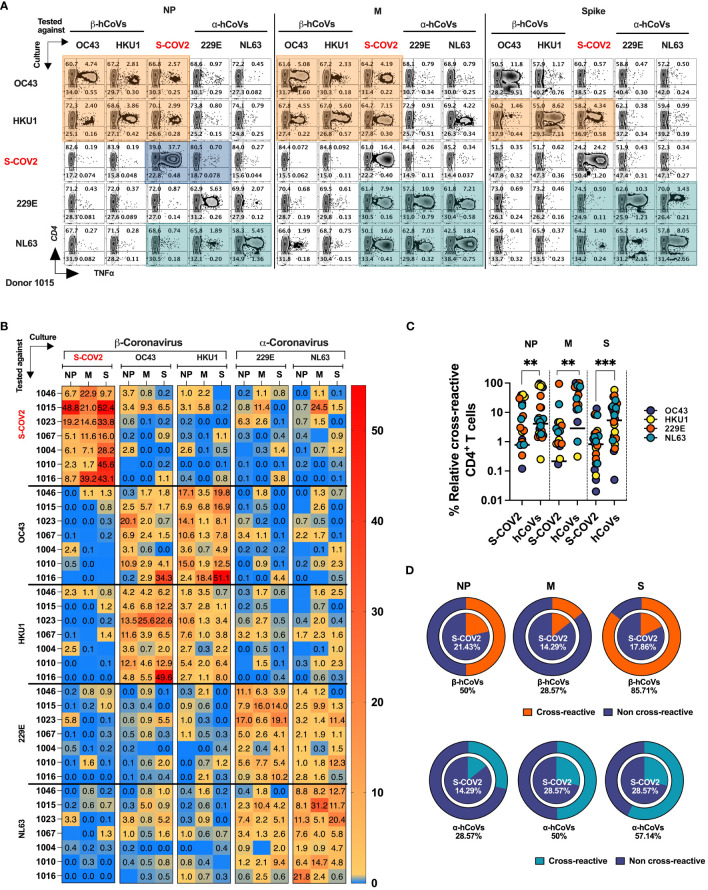
T cell cross-reactivity between SARS-CoV-2 and hCoVs. Comprehensive T cell cross-reactivity analysis among two α-hCoVs, two β-hCoVs and SARS-CoV-2 against three immunodominant antigens S, M and NP. PBMCs from each donor were stimulated by either S1 & S2 or M & NP peptide libraries against all 5 viruses generating 10 culture conditions per donor. Each culture was challenged by its counterpart antigen from other four viruses along with cognate antigen creating a cross reactivity matrix. Orange boxes represent cross-reactivity of β-hCoVs, green boxes represent cross-reactivity of α-hCoVs while blue boxes represent that of SARS-CoV-2. **(A)** Representative zebra plots showing full cross-reactivity matrix between five coronaviruses against three antigens. **(B)** Heatmap showing relative cross-reactivity in CD4^+^ T cell compartment. **(C)** Comparison of relative cross-reactivity between SCoV2 and hCoVs against M, NP, and S protein among CD4^+^ T cell compartment. Horizontal lines indicate the mean value. **(D)** Relative frequency (%) of donors displaying cross-reactivity between *ex vivo* expanded T cell populations specific for SARS-CoV-2 (SCoV2; inner circle) and indicated α- and β-hCoVs (outer circles) against M, NP, and S protein within the CD4^+^ T cell compartment. Statistically significant differences of reactivity were determined by Mann-Whitney test. **P < 0.01, ***P < 0.001.

To directly evaluate the degree of T cell cross-reactivity against immunodominant antigens from SARS-CoV-2 and hCoVs, we primed and expanded PBMCs from donors (n=7, five HCW and two IP subjects) with documented history of COVID-19 using the panel of Spike (S1 plus S2), NP and M pepmixes derived from each of the coronavirus included in the study. The resulting T cell populations from each donor were tested against the cognate peptide pool initially used for priming and for cross-reactivity with the counterpart antigens from the other members of CoV family, generating a matrix map of potential cross-reactivity. A schematic diagram outlining the experimental strategy is provided in **Supplementary Figure S5A**. A representative example (#1015) is shown in **Figure 6A**, while **Figure 6B** provides a heatmap distribution summarizing the antigen-specific CD4^+^ T cell reactivity against all five viruses for all analyzed donors. The horizontal axis represents the virus and antigen used for priming and expanding the T cell cultures, while the vertical axis represents the virus and antigen used for stimulating the T cell cultures. Subject #1015 is a patient with active multiple myeloma who developed symptomatic COVID-19 at the beginning of the pandemic (March 2020) following a cycle of chemotherapy and subsequently underwent autologous SCT. Another IP Subject #1023 included in this experiment is a patient with history of erythropoietic protoporphyria (EPP), failed liver and hematopoietic stem cell transplant followed by the second liver and SCT rescue who experienced COVID-19 and was previously described in a case report (30).

The *ex vivo* expanded T cells from all donors vigorously recognized cognate antigens of SARS-CoV-2 and the majority of hCoVs. Cross-recognition was clearly present between counterpart antigens from the closely related non-SARS α- and β-hCoV (NL63 vs 229E and OC43 vs. HKU1, respectively), as anticipated by the relatively high degree of homology between these viruses In participant #1015, T cells specific to β-hCoVs (OC43 and HKU1) NP and M antigens also recognized NP and M antigens derived from SARS-CoV-2, indicating a degree of cross-reactivity (**Figure 6A**, orange boxes). Moreover, T cells specific to HKU1 S antigen also displayed cross-recognition of SARS-CoV-2 S antigen. This cross-reactive pattern was similarly observed in both α-hCoVs (229E and NL63, green boxex) specific T cell responses directed against M antigen, and to a lesser extent against NL63 S and NP antigens, demonstrating some discernible cross-reactivity with SARS-CoV-2. Notably, the donor displayed a robust and highly specific response against NP, M, and S antigens of SARS-CoV-2 (comprising 49.15%, 21.19%, and 50% CD4+ TNF-α+ T cells, respectively). However, these T cells showed limited capability to cross-recognize analogous antigens from other hCoVs, as depicted in the upper panel of **Figure 6A** (illustrated by blue boxes). Extensive cross-reactivity between two related α-hCoVs (229E vs NL63) and two β-hCoVs (OC43 and HKU1) was seen in samples from all donors, while SARS-CoV-2 responses displayed relatively little cross-recognition (**Figure 6B**). These observations suggest that upon resolution of COVID-19 the emerging T cell responses against SARS-CoV-2 NP, M and S antigens are highly focused and distinct from the responses mounted against the related non-SARS α and β hCoVs. However, upon priming with non-SARS hCoV antigens a degree of non-reciprocal cross-reactivity against SARS-CoV-2 antigens may be observed in the same subjects, that is likely targeting different non-dominant epitopes (**Figures 6A, B**). Consequently, T cells expanded using antigens from non-SARS hCoVs showed significantly higher relative cross-reactivity towards the counterpart SARS-CoV-2 targets while SARS-CoV-2-specific T cell cultures were relatively less cross-reactive with non-SARS-CoV-2 antigens in the same donor (**Figure 6C**). Additionally, more donors displayed cross-reactivity (**Figure 6D**) in T cell cultures primed initially with non-SARS β-hCoV and α-hCoVs antigens (external pie charts) as compared to only few samples displaying cross reactivity in the opposite direction (internal pie charts). Overall, the data indicates a relatively high degree of cross-reactivity between T cells specific for related non-SARS hCoV family members, while variable and non-reciprocal cross-reactivity might be seen with the antigens of SARS-CoV-2. These observations might be of practical use when designing strategies aimed at development of universal anti-CoV vaccination or cell-based immunotherapy.

### Distinct T cell receptor repertoire targets SARS-CoV-2 and common hCoV antigens in survivors of COVID-19

Based on the observed cross-reactivity pattern in flow cytometry, we hypothesized that the focused T cell repertoire directed against SARS-CoV-2 antigens emerges following the resolution of the infection, with relatively little overlap with the repertoire directed against the antigens from non-SARS α- and β-hCoVs. Conversely, T cells primed to recognize hCoV antigens might be capable of targeting some epitopes from SARS-CoV-2, but this cross-recognition favors distinct antigens and is dominated by non-overlapping clonotypes. To test this hypothesis, we carried out a comprehensive analysis of the TCRβ repertoire of T cells targeting NP, M, and S antigens of SARS-CoV-2, as well as their hCoV counterparts. We studied two individuals who had survived COVID-19: IP subject #1023 (as described earlier) and a healthcare worker (HCW) #1046, who was otherwise healthy.

To further characterize this phenomenon, we carried out in-depth analysis of the TCRβ repertoire of T cells specific for SARS-CoV-2 NP, M and S antigens and their hCoV counterparts in two survivors of COVID-19: IP subject #1023 (previously described) and an otherwise healthy HCW #1046. The strategy used for TCR sequencing of antigen-specific T cells is outlined as a schematic diagram in **Supplementary Figure S5B**.

Virus-specific T cell cultures were generated from both donors using the M, NP and S pepmixes from all 5 viruses (OC43, HKU1, SCoV2, 229E and NL63). Antigen-specific T cells were FACS-sorted using TNF-α cytokine capture (**Figure S5C**) (47), allowing for analysis of highly purified antigen-specific T cells with known antigenic reactivity and largely devoid of passenger T cells. The CDR3 TCRβ repertoires of the isolated M, NP and S-specific T cells targeting each virus were analyzed using Adaptive Biotechnologies’ ImmunoSEQ platform.

**Figure 7:**
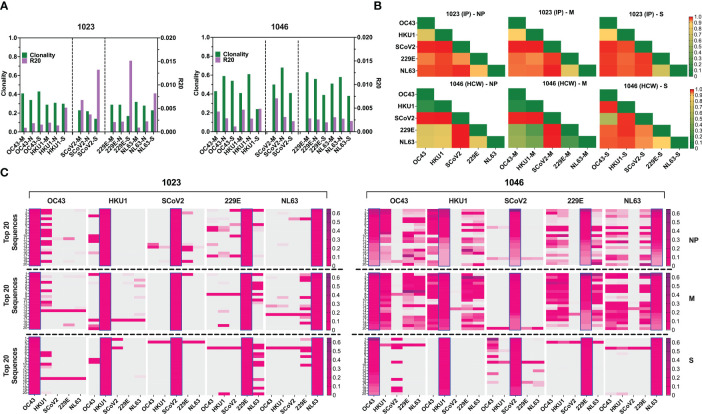
TCR repertoire analysis shows focused response to SARS-CoV-2 with few cross-reactive TCRs. **(A)** Clonality and R20 values within each antigen-specific T cell populations generated using three immunodominant antigens S, M and NP from two α-hCoVs, two β-hCoVs and SARS-CoV-2 virus. **(B)** TCR repertoire similarity between antigen-specific populations recognizing NP, M and S targets from all 5 tested CoVs measured by JSD index (ranges 0-1) and shown in heatmaps. **(C)** Heatmaps showing the top 20 dominant clones ranked by frequencies within each designated sample highlighted in blue rectangular within each panel. Their cross-reactivity with other CoVs is reflected by the co-presence of the same sequence in the same row in donor #1023 (Left panel), Donor 1046 (Right panel).

The number of unique TCRβ sequences, defined by CDR3+V+ J at the amino acid level, identified from each subset ranged from 462 to 2849 for donor #1023 (**Figure S5D**, left panel), and 214 to 1438 for donor #1046, respectively (**Figure S5D**, right panel). The TCRβ repertoire specific for each antigen displayed high clonality scores (0.14 to 0.68) and low R20 score (range 0.00089246 to 0.015151), indicating dominant contribution of clonal expansion with restricted diversity (**Figure 7A**). Top 10 most prevalent SARS-CoV-2-specific T cell clones against different antigen epitopes from subject #1023 represented 11.89 to 21.44% of all sequences identified. In Donor #1046 this fraction was 89.53, 82.03, 61.01% of NP, M and S-specific sequences respectively (**Figure S6A**) with the most abundant NP-derived sequence representing approximately 60% of the repertoire, indicating a highly reactive clonotype.

To estimate the possible presence of the cross-reactive TCRs, we performed global similarity analysis of TCRβ repertoire specific for each antigen of each CoV using Jensen-Shannon divergence (JSD) index. Lower JSD scores (indicating higher repertoire convergence) were seen between the corresponding NP- M- and S- reactive T cells specific for closely related 229E/NL63 α-hCoVs and OC43/HKU1 β-hCoVs (**Figure 7B**). There was also some degree of repertoire similarities between populations targeting the corresponding antigens of α- and β-hCoVs, especially in #1046 NP and M samples (JSD ranges from 0.267747 to 0.80066). In the same subject a high similarity was observed between OC43-S and SARS-CoV-2-S specific T cell repertoire (JSD=0.585714). Lower similarity was seen in both tested donors when SARS-CoV-2-specific repertoire was compared with the repertoires specific for the corresponding antigens from α- or β-hCoVs, as predicted upon *in vitro* antigen cross-reactivity pattern.

We also analyzed the clonal composition of the T cell repertoire. In both donors, the majority of isolated TCR sequences were unique for each antigen of each CoVs, as shown in the global Venn diagrams (**Figure S6B**), but a small number of TCR sequences shared among all five tested repertoires was seen (ranges 8-30). The analysis of the top 20 most abundant CDR3 sequences specific for each viral antigen (**Figure 7C**) revealed certain dominant clonotypes shared between both α- and both β-hCoVs. We also found abundant TCR clonotypes specific for all four non-SARS hCoVs, especially among NP and M-reactive population in subject #1046. However, in both donors there were relatively few dominant TCR sequences derived from the SARS-CoV-2 specific population that were shared with other hCoVs, with the exception of #1023 NP antigen overlapping with some sequences from 229E hCoV (**Figure 7C**, left panel). In #1046 the overlap was seen predominantly with S antigens of OC43 hCoV (**Figure 7C**, right panel) where the most abundant CDR3 sequence (53.1%) was also the most abundant clonotype within the SARS-CoV-2-S, representing 14.1% of total CDR3 repertoire, suggestive of possible cross-reactivity. Furthermore, multiple TCRs specific to SARS-CoV-2 antigens identified from both donors were found in the Adaptive Biotechnologies’ ImmuneCODE database (35) (**Figure S6C**), implying shared/public anti-viral TCRs that are frequently present in the general population. Interestingly, several of the TCR sequences isolated by us from the non-SARS hCoV cultures were found in the ImmuneCODE database. These TCR sequences may represent public clonotypes specific to “common cold” hCoVs antigens, rather than uniquely induced by the COVID-19-related antigens. Taken together, a highly focused TCR repertoire emerges in COVID-19 survivors that has relatively little overlap with T cells induced upon exposure to M, NP and S antigens from non-SARS hCoV family members. In contrast, there is a relatively high frequency of shared TCRs specific to α or β-hCoVs expressed in T cells responding to the closely related members of each hCoV subfamily, as predicted by the high degree of functional cross-reactivity.

### Generation of the universal multi-CoV-specific T cells for adoptive immunotherapy or prophylaxis of COVID-19 and hCoV infections

**Figure 8:**
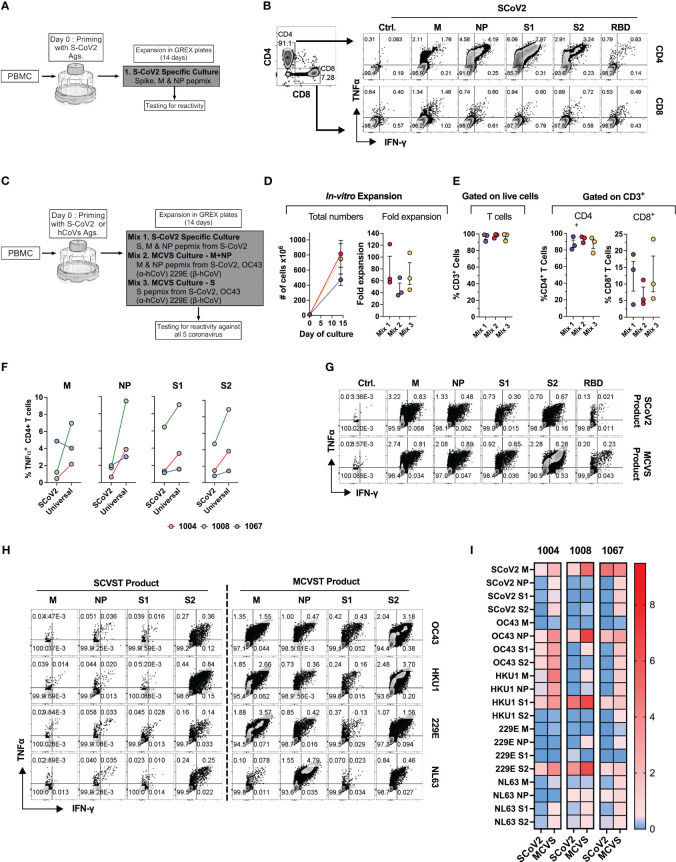
Novel strategy to generate multi-coronavirus specific T cell (MCVST) product for adoptive T cell therapy. **(A)** Schematic diagram showing strategy to generate clinical grade SARS-CoV-2 specific T cells (SCVST) for adaptive cell therapy using G-REX bioreactors. **(B)** Representative zebra plots showing reactivity of SCVST product in CD4 and CD8 T cell compartments. **(C)** Schematic diagram showing strategy to generate multi-coronavirus specific (MCVS) T cells. **(D)** *Ex vivo* expansion (total viable cells) and fold change of the clinical-scale products generated in G-REX flasks in 14 days. **(E)** Percentage of CD3^+^, CD4^+^ and CD8^+^ T cells in the final product. **(F)** Comparison of reactive CD4^+^ T cell frequency between SCVST and MCVST products against indicated antigen **(G)** Representative zebra plots demonstrating reactivity of SCVST product and MCVST product from the same donor. **(H)** Representative zebra plots showing reactivity of SARS-CoV-2 specific T cell product and MCVST product against the indicated human hCoV antigens. **(I)** Heatmap showing frequency antigen-specific CD4^+^ T cell against indicated antigens (S1, S2, M, NP) of two α-hCoVs (229E, NL63), two β-hCoVs (OC43, HKU1) and SARS-CoV-2.

Finally, based on the *ex vivo* priming strategy, we hypothesized that it would be feasible to generate SARS-CoV-2-specific T (SCVST) cells using a clinical-grade procedure compatible with current Good Manufacturing Practice (cGMP) methodology. First, the dedicated COVID-19 products were generated by culturing PBMCs from COVID-exposed donors stimulated with SARS-CoV-2 M, NP and S1/S2 peptide mixes pulsed into irradiated autologous PBMCS as antigen presenting cells (APCs). Cultures were maintained for 14 days in G-Rex gas-permeable containers (**Figure 8A**). The resulting SCVST cells displayed robust expansion (not shown) and predominantly contained CD4^+^ T cells (>90%). Upon *in vitro* stimulation, the SCVSTs recognized all SARS-CoV-2 antigens, as well as the S1 fragment representing Receptor Binding Domain (RBD) of the virus (**Figure 8B**).

Next, based on the cross-reactivity pattern and TCR sequencing results seen in COVID+ donors (**Figures 6**, **7**), we hypothesized that a broadly applicable universal multi-hCoVs-specific T (MCVST) cell product might be generated by using SARS-CoV-2 antigens supplemented with counterpart peptide mixes from a single α-hCoV and a single β-hCoV. To test this hypothesis, the parallel cultures were initiated using M, N and S1/S2 antigens of SARS-CoV-2 (Mix 1: SCVST cells), a culture primed with M and NP antigens from SARS-CoV-2, 229E α-hCoV and OC43 β-hCoV (Mix 2: MCVST cells-M+NP) and a culture primed with S1 and S2 antigens form SARS-CoV-2, 229E α-hCoV and OC43 β-hCoV (Mix 3: MCVST cells-S) (**Figure 8C**). All cultures expanded robustly (range 35.76-121.88 -fold) yielding 3.6x10^8^ to 1028.6X10^8^ viable cells (**Figure 8D**) including over 90% CD3^+^ T cells. Of those over 75% were CD4^+^ (range 76.3 to 95.8%) while CD8^+^ T cells comprised 3.8% to 23.5% of CD3^+^ T cells (**Figure 8E**).

To assess the anti-coronavirus response of SCVST and MCVST products, we measured the frequency of antigen-specific T cells against all five viruses used in the study. Intriguingly, the universal MCVST cultures displayed higher frequencies of SARS-CoV-2 M, NP, and S1/S2-reactive cells compared to the focused SCVST cultures primed with only SARS-CoV-2 antigens. However, these differences were not statistically significant (**Figure 8F**). **Figure 8G** shows representative zebra plots that depict a comparative analysis of the SCoV2-specific response within SCVST and MCVST products. Remarkably, the final MCVST products also demonstrated a significant capability to recognize non-SARS hCoVs, such as NL63 and HKU1 hCoVs, which were not initially used for priming (**Figures 8H, I**). Comparison of the universal coronaviral response between SCVST and MCVST products derived from a single donor is depicted in **Figure 8H**. These findings shed light on the robustness and broad-spectrum reactivity of the MCVST products, indicating their potential applicability in combating various coronaviruses. The overall reactivity against all three antigens (S, M, NP) of all 5 CoVs tested across 3 donors is shown as a heatmap (**Figure 8I**). In summary, priming with a combination of antigens from SARS-CoV-2 supplemented with one α-hCoV and one β-hCoV antigen yields MCVST cells with an enhanced ability to target SARS-CoV-2, while also inducing broad reactivity against “common cold” hCoVs that frequently affect vulnerable immunocompromised patients. This strategy provides proof-of-concept for generating universal MCVST cells capable of protecting or treating current and future variants and emerging diseases caused by human and zoonotic CoVs.

## Discussion

T cell-mediated responses are essential for efficacy of host defense against viral infections. *Ex vivo* expanded virus-specific T (VST) cells have been successfully used to target common refractory viral infections in immunocompromised patients (48, 49). There is growing evidence that T cell immunity is also critical for protection against the severe complications of SARS-CoV-2 infection (13, 50, 51). Notably, patients with inborn defects in B cell functionality frequently experienced only mild course of COVID-19, suggesting that cellular immunity is sufficient for effective host defenses (52, 53). Furthermore, immunocompromised cancer patients were relatively protected from severe COVID-19 if T cell function was preserved (18). Importantly, evidence from the SARS-CoV1 outbreak and emerging findings from COVID-19 indicate that a long-lived T cell memory is induced in survivors (19, 21, 54).

Seasonal infections with “common cold” hCoVs are highly prevalent but remain relatively understudied (9, 55). Intriguingly, emerging data suggest that pre-existing cross-reactive immunological memory induced by prior infections with endemic hCoVs may ameliorate the severity of the subsequent infection with SARS-CoV-2 (25). Understanding the T cell response against SARS-CoV-2 and related hCoVs is critical for the development of adoptive transfer strategies intended for vulnerable cancer and other immunocompromised patients with impaired cellular immunity. Here we tested T cell immune responses against immunodominant antigens (S, M, NP) from SARS-CoV-2 and counterpart antigens from α-hCoVs (229E and NL63) and β-hCoVs (OC43 and HKU1) in exposed and unexposed healthy volunteers and immunocompromised survivors of COVID-19, using a custom panel of overlapping peptide libraries including commonly investigated S1 and S2 proteins, as well as less-studied NP and M proteins from each virus. We used the microscale priming/expansion strategy similar to clinical-grade manufacturing of VST cells, incorporating a period of specific expansion and enrichment prior to antigenic testing, enabling unequivocal detection of antigen-specific T cell repertoires. Our approach requires a relatively small number of PBMCs, is highly sensitive and allows for functional determination of the immunocompetence upon re-challenge with viral antigens, permitting for unbiased determination of potential cross-reactivity. We have previously used this method to measure the baseline T cell immunity of patients with Progressive Multifocal Leukoencephalopathy (PML) against John Cunningham (JC) Polyomavirus antigens LT and VP1 as a marker to predict the likelihood clinical response to PD-1 blockade (39). In contrast, the conventional methods (ELISPOT or AIM) have the advantage of estimating the frequency and phenotype of pathogen-specific T cells directly in peripheral blood, but they require large numbers of cells and may be less sensitive when analyzing low-frequency events (36, 56).

Consistent with previous reports (13, 38), we observed T cells responding to SARS-CoV-2 antigens in many unvaccinated/unexposed individuals with no documented history of COVID-19, but these responses were highly variable in magnitude and generally favored Spike antigens. Vigorous anti-S1/S2 T cell responses emerged post-vaccination even in immunocompromised subjects, whereas in survivors of COVID-19, broad T cell repertoire targeting Spike and non-spike antigens, such as M and NP, of SARS-CoV-2 emerged. Importantly, potent T cell reactivity was seen not only among the otherwise healthy survivors of COVID-19, but also among high-risk patients with a history of hematological malignancy, SCT and SOT, who survived the infection. However, most of the analyzed subjects, including those in the immunocompromised cohort, experienced a relatively mild course of COVID-19 and only one of them required hospitalization. Similarly, our vaccinated IP group included only few very-high risk individuals undergoing active chemotherapy immediately after the transplant.

The anti-SARS-CoV-2 responses were predominantly and consistently seen within the CD4^+^ Th cells and retained polyfunctionality as evidenced by production of TNF-α, IFN-γ, GZMB and IL-2. CD8^+^ T cell reactivity was more variable between donors. This might be partially due to the inherent tendency of the longer (15-aminoacid) peptides used for priming to stimulate T cells via class II MHC (57, 58). However, CD8^+^ T cell reactivity against other viral antigens from BK, AdV and EBV was often detectable in the same assay, suggesting that the pattern of recognition is virus- and donor-specific (14) CD4^+^ T cells might be critical for favorable outcomes of COVID-19 (14) and for vaccine responses (59). The relative magnitude of T cell responses against non-Spike antigens was comparable to or higher than the reactivities against S1 and S2 in survivors of COVID-19. However, the *in vivo* contribution of S, M and NP-specific T cell responses in protection against SARS-CoV-2 is unknown. Our data suggest the potential feasibility of targeting non-Spike antigens in the new generation of vaccines (56, 60). In fact, it has been previously shown that certain class I-restricted NP responses correlate with less viral replication and highly favorable outcomes (61).

Furthermore, our observations indicate that CD4^+^ T cells induced upon priming with the ancestral SARS-CoV-2 S1 and S2 epitopes largely retained the ability to recognize mutated antigens representing variants of the virus that emerged during the evolution of the COVID-19 pandemic. This underscores the more promiscuous nature of class II-restricted T cell responses capable of activation by altered peptide ligands. This cross-reactivity was seen in both COVID-19 survivors and in the vaccinated unexposed group. However, in the case of the *omicron* variant harboring over 30 mutations in the Spike protein, *ex vivo* expanded T cells initially primed using the ancestral or *omicron* S1/S2 retained less than 50% cross-reactivity upon testing against their counterparts.

Thus, previous infection and/or vaccination induces broad immunological memory capable of recognizing S1/S2 proteins from novel variants of SARS-CoV-2, but at least partial loss of protection is likely against highly divergent variants such as *omicron*. However, exploiting and/or inducing additional M and NP-directed T cell immunity might provide additional layer of protection against the emerging threats from future variants if anti-Spike cross-reactivity is diminished.

In parallel, we studied reactivity against the endemic hCoV, observing immune responses in the majority of healthy and immunocompromised subjects. Interestingly, COVID-19 survivors displayed a broader reactivity pattern against the endemic viruses, suggesting possible induction of cross-reactive immunity upon resolution of COVID-19 that is increasingly recognized in literature (62, 63). *Ex vivo* expanded T cells specific for SARS-CoV-2 S, M and NP antigens and the equivalent antigens from hCoVs were further tested for cross-reactivity. The most vigorous reciprocal cross-recognition was seen between T cells initially primed to recognize closely related α-hCoV (229E vs NL63) and β-hCoV (OC43 vs. HKU1), while cross-reactivity between T cells specific for α and β antigens was less prominent. Cross-recognition of counterpart antigens from non-SARS hCoVs and SARS-CoV-2 was seen in some donors, often displaying unidirectional/non-reciprocal pattern. Namely, T cells initially primed to recognize hCoV antigens were more cross-reactive, whereas priming with SARS-CoV-2 antigens induced minimally cross-reactive populations, likely indicating focused immunological memory skewed towards non-shared dominant epitopes (62). TCR sequencing of highly purified antigen-specific T cells isolated from two COVID-19 survivors elucidates the basis of this observation, revealing dominant clonotypes shared between α or β-hCoV-reactive populations, while SARS-CoV-2-specific T cells contained distinct repertoires. However, this phenomenon might warrant more extensive characterization to draw a broader conclusion, as some universal anti-CoV clonotypes were also identified (data not shown). Finally, based on our cross-reactivity observations, we tested feasibility of creating universal Multi-CoV-specific T cells opening the prospect of application in adoptive cellular therapy or prophylaxis in SCT patients, or immunocompromised patients with protracted COVID-19 (29, 64). Concurrent priming of PBMCs with SARS-CoV-2 antigens and counterpart antigens from a single α-hCoV and a single β-hCoV induced a population with enhanced capacity to target SARS-CoV-2 antigens and with potent activity against all four endemic hCoVs. This synergy further underscores the interplay between the immune responses directed against multiple related members of the CoV family.

In summary, to our knowledge, this is the first study to comprehensively investigate the cross-reactivity of SARS-CoV-2 and hCoVs against all three major immunogenic antigens of coronaviruses, namely S, M and NP. Our data supports the hypothesis that a broadly-specific universal anti-CoV T cell-directed vaccines and cellular therapy products are feasible as preventive or rescue immunotherapies.

## Data availability statement

The datasets presented in this study can be found in online repositories. The names of the repository/repositories and accession number(s) can be found below: https://clients.adaptivebiotech.com/pub/mithil-2023-fi, SM2023FI.

## Ethics statement

The studies involving humans were approved by Healthy volunteers, primarily healthcare workers and associated individuals, with or without COVID-19 exposure, as well as immunocompromised patients were included after informed consent under an IRB approved protocol (IRB-AAAT1895). The studies were conducted in accordance with the local legislation and institutional requirements. The participants provided their written informed consent to participate in this study.

## Author contributions

MKS, EM, and PM designed research studies, MKS, EM conducted experiments, MKS, EM, JP acquired data, MKS, EM, JF analyzed data, AA, HC coordinated sample collection from subjects, MS, EM, JF and PM wrote the manuscript, RC, PK, MSM, MP, MYM, VG and MS edited manuscript. All authors contributed to the article and approved the submitted version.

## References

[1] Organization, W.H. WHO Coronavirus (COVID-19) Dashboard. Available at: https://covid19.who.int/.

[2] Liu J Xie W Wang Y Xiong Y Chen S Han J. A comparative overview of COVID-19, MERS and SARS: Review article. Int J Surg (2020) 81:1–8. doi: 10.1016/j.ijsu.2020.07.032 32730205 PMC7382925

[3] Breban R Riou J Fontanet A. Interhuman transmissibility of Middle East respiratory syndrome coronavirus: estimation of pandemic risk. Lancet (2013) 382(9893):694–9. doi: 10.1016/S0140-6736(13)61492-0 PMC7159280 23831141

[4] Zumla A Hui DS Perlman S. Middle East respiratory syndrome. Lancet (2015) 386(9997):995–1007. doi: 10.1016/S0140-6736(15)60454-8 26049252 PMC4721578

[5] Leung GM Hedley AJ Ho L-M Chau P Wong IOL Thach TQ. The epidemiology of severe acute respiratory syndrome in the 2003 Hong Kong epidemic: an analysis of all 1755 patients. Ann Intern Med (2004) 141(9):662–73. doi: 10.7326/0003-4819-141-9-200411020-00006 15520422

[6] Peiris JSM Chu C-M Cheng VC-C Chan K Hung I Poon LL. Clinical progression and viral load in a community outbreak of coronavirus-associated SARS pneumonia: a prospective study. Lancet (2003) 361(9371):1767–72. doi: 10.1016/S0140-6736(03)13412-5 PMC7112410 12781535

[7] Gaunt ER Hardie A Claas ECJ Simmonds P Templeton KE. Epidemiology and clinical presentations of the four human coronaviruses 229E, HKU1, NL63, and OC43 detected over 3 years using a novel multiplex real-time PCR method. J Clin Microbiol (2010) 48(8):2940–7. doi: 10.1128/JCM.00636-10 PMC2916580 20554810

[8] Corman VM Muth D Niemeyer D Drosten C. Chapter Eight - Hosts and Sources of Endemic Human Coronaviruses. In: Kielian M, Mettenleiter TC, Roossinck MJ, editors. (Academic Press). Adv Virus Res (2018) 100:163–88. doi: 10.1016/bs.aivir.2018.01.001 PMC7112090 29551135

[9] Jo KJ Choi SH Oh CE Kim H Choi BS Jo DS. Epidemiology and clinical characteristics of human coronaviruses-associated infections in children: A multi-center study. Front Pediatr (2022) 10:877759. doi: 10.3389/fped.2022.877759 35498812 PMC9039334

[10] Masse S Capai L Villechenaud N Blanchon T Charrel R Falchi A. Epidemiology and clinical symptoms related to seasonal coronavirus identified in patients with acute respiratory infections consulting in primary care over six influenza seasons (2014-2020) in France. Viruses (2020) 12(6). doi: 10.3390/v12060630 PMC7354536 32532138

[11] Ogimi C Greninger AL Waghmare AA Kuypers JM Shean RC Xie H. Prolonged shedding of human coronavirus in hematopoietic cell transplant recipients: risk factors and viral genome evolution. J Infect Dis (2017) 216(2):203–9. doi: 10.1093/infdis/jix264 PMC5853311 28838146

[12] Vardhana S Baldo L Morice WG Wherry EJ. Understanding T cell responses to COVID-19 is essential for informing public health strategies. Sci Immunol (2022) 7(71):eabo1303. doi: 10.1126/sciimmunol.abo1303 35324269 PMC10344642

[13] Grifoni A Weiskopf D Ramirez SI Mateus J Dan JM Moderbacher CR. Targets of T cell responses to SARS-coV-2 coronavirus in humans with COVID-19 disease and unexposed individuals. Cell (2020) 181(7):1489–1501.e15. doi: 10.1016/j.cell.2020.05.015 32473127 PMC7237901

[14] Tarke A Potesta M Varchetta S Fenoglio D Iannetta M Sarmati L. Early and polyantigenic CD4 T cell responses correlate with mild disease in acute COVID-19 donors. Int J Mol Sci (2022) 23(13). doi: 10.3390/ijms23137155 PMC9267033 35806161

[15] Rydyznski Moderbacher C Ramirez SI Dan JM Grifoni A Hastie KM Weiskopf D. Antigen-specific adaptive immunity to SARS-coV-2 in acute COVID-19 and associations with age and disease severity. Cell (2020) 183(4):996–1012.e19. doi: 10.1016/j.cell.2020.09.038 33010815 PMC7494270

[16] Le Bert N Clapham HE Tan AT Chia WN Tham CY Lim JM. Highly functional virus-specific cellular immune response in asymptomatic SARS-CoV-2 infection. J Exp Med (2021) 218(5). doi: 10.1084/jem.20202617 PMC7927662 33646265

[17] Sekine T Perez-Potti A Rivera-Ballesteros O Strålin K Gorin J-B Olsson A. Robust T cell immunity in convalescent individuals with asymptomatic or mild COVID-19. Cell (2020) 183(1):158–168.e14. doi: 10.1016/j.cell.2020.08.017 32979941 PMC7427556

[18] Bange EM Han NA Wileyto P Kim JY Gouma S Robinson J. CD8+ T cells contribute to survival in patients with COVID-19 and hematologic cancer. Nat Med (2021) 27(7):1280–9. doi: 10.1038/s41591-021-01386-7 PMC8291091 34017137

[19] Jung JH Rha M-S Sa M Choi HK Jeon JH Seok H. SARS-CoV-2-specific T cell memory is sustained in COVID-19 convalescent patients for 10 months with successful development of stem cell-like memory T cells. Nat Commun (2021) 12(1):4043. doi: 10.1038/s41467-021-24377-1 34193870 PMC8245549

[20] Zhao J Zhao J Mangalam AK Channappanavar R Fett C Meyerholz DK. Airway memory CD4(+) T cells mediate protective immunity against emerging respiratory coronaviruses. Immunity (2016) 44(6):1379–91. doi: 10.1016/j.immuni.2016.05.006 PMC4917442 27287409

[21] Ng O-W Chia A Tan AT Jadi RS Leong HN Bertoletti A. Memory T cell responses targeting the SARS coronavirus persist up to 11 years post-infection. Vaccine (2016) 34(17):2008–14. doi: 10.1016/j.vaccine.2016.02.063 PMC7115611 26954467

[22] Meyerholz DK Perlman S. Does common cold coronavirus infection protect against severe SARS-CoV-2 disease? J Clin Invest (2021) 131(1). doi: 10.1172/JCI144807 PMC7773392 33216734

[23] Sagar M Reifler K Rossi M Miller NS Sinha P White LF. Recent endemic coronavirus infection is associated with less-severe COVID-19. J Clin Invest (2021) 131(1). doi: 10.1172/JCI143380 PMC7773342 32997649

[24] Mateus J Grifoni A Tarke A Sidney J Ramirez SI Dan JM. Selective and cross-reactive SARS-CoV-2 T cell epitopes in unexposed humans. Science (2020) 370(6512):89–94. doi: 10.1126/science.abd3871 32753554 PMC7574914

[25] Kundu R Narean JS Wang L Fenn J Pillay T Fernandez ND. Cross-reactive memory T cells associate with protection against SARS-CoV-2 infection in COVID-19 contacts. Nat Commun (2022) 13(1):80. doi: 10.1038/s41467-021-27674-x 35013199 PMC8748880

[26] Araf Y Akter F Tang YD Fatemi R Parvez MSA Zheng C. Omicron variant of SARS-CoV-2: genomics, transmissibility, and responses to current COVID-19 vaccines. J Med Virol (2022) 94(5):1825–32. doi: 10.1002/jmv.27588 PMC9015557 35023191

[27] Liu L Iketani S Guo Y Chan JFW Wang M Liu L. Striking antibody evasion manifested by the Omicron variant of SARS-CoV-2. Nature (2022) 602(7898):676–81. doi: 10.1038/s41586-021-04388-0 35016198

[28] Grabowski F Kochańczyk M Lipniacki T. The spread of SARS-coV-2 variant omicron with a doubling time of 2.0&ndash;3.3 days can be explained by immune evasion. Viruses (2022) 14(2):294. doi: 10.3390/v14020294 35215887 PMC8875689

[29] Conway SR Keller MD Bollard CM. Cellular therapies for the treatment and prevention of SARS-CoV-2 infection. Blood (2022) 140(3):208–21. doi: 10.1182/blood.2021012249 PMC8896869 35240679

[30] Soni M Migliori E Assal A Chan HT Ciubotariu R Pan JB. Development of T-cell immunity in a liver and hematopoietic stem cell transplant recipient following coronavirus disease 2019 infection. Cytotherapy (2021) 23(11):980–4. doi: 10.1016/j.jcyt.2021.05.005 PMC8165078 34183244

[31] Langerak AW Groenen PJ Jm van Krieken JH van Dongen JJ. Immunoglobulin/T-cell receptor clonality diagnostics. Expert Opin Med Diagn (2007) 1(4):451–61. doi: 10.1517/17530059.1.4.451 23496353

[32] Heberle H Meirelles GV da Silva FR Telles GP Minghim R. InteractiVenn: a web-based tool for the analysis of sets through Venn diagrams. BMC Bioinf (2015) 16:169. doi: 10.1186/s12859-015-0611-3 PMC4455604 25994840

[33] Fu J Zuber J Shonts B Obradovic A Wang Z Frangaj K. Lymphohematopoietic graft-versus-host responses promote mixed chimerism in patients receiving intestinal transplantation. J Clin Invest (2021) 131(8). doi: 10.1172/JCI141698 PMC8062082 33630757

[34] Snyder TM Gittelman RM Klinger M May DH Osborne EJ Taniguchi R. Magnitude and dynamics of the T-cell response to SARS-coV-2 infection at both individual and population levels. medRxiv (2020). doi: 10.1101/2020.07.31.20165647 PMC11747429 39840037

[35] Nolan S Vignali M Klinger M Dines JN Kaplan IM Svejnoha E. A large-scale database of T-cell receptor beta (TCRbeta) sequences and binding associations from natural and synthetic exposure to SARS-CoV-2. Res Sq (2020). doi: 10.21203/rs.3.rs-51964/v1 PMC11873104 40034696

[36] Reiss S Baxter AE Cirelli KM Dan JM Morou A Daigneault A. Comparative analysis of activation induced marker (AIM) assays for sensitive identification of antigen-specific CD4 T cells. PloS One (2017) 12(10):e0186998. doi: 10.1371/journal.pone.0186998 29065175 PMC5655442

[37] Dan JM Lindestam Arlehamn CS Weiskopf D da Silva Antunes R Havenar-Daughton C Reiss SM. A cytokine-independent approach to identify antigen-specific human germinal center T follicular helper cells and rare antigen-specific CD4+ T cells in blood. J Immunol (2016) 197(3):983–93. doi: 10.4049/jimmunol.1600318 PMC4955771 27342848

[38] Bacher P Rosati E Esser D Martini GR Saggau C Schiminsky E. Low-avidity CD4(+) T cell responses to SARS-coV-2 in unexposed individuals and humans with severe COVID-19. Immunity (2020) 53(6):1258–1271 e5. doi: 10.1016/j.immuni.2020.11.016 33296686 PMC7689350

[39] Cortese I Muranski P Enose-Akahata Y Ha SK Smith B Monaco M. Pembrolizumab treatment for progressive multifocal leukoencephalopathy. N Engl J Med (2019) 380(17):1597–605. doi: 10.1056/NEJMoa1815039 30969503

[40] Hachmann NP Miller J Collier A-rY Ventura JD Yu J Rowe M. Neutralization escape by SARS-coV-2 omicron subvariants BA. 2.12. 1, BA. 4, and BA. 5. N Engl J Med (2022) 387:86–8. doi: 10.1101/2022.05.16.22275151 PMC9258748 35731894

[41] Cele S Jackson L Khoury DS Khan K Moyo-Gwete T Tegally H. Omicron extensively but incompletely escapes Pfizer BNT162b2 neutralization. Nature (2022) 602(7898):654–6. doi: 10.1038/s41586-021-04387-1 PMC8866126 35016196

[42] Planas D Saunders N Maes P Guivel-Benhassine F Planchais C Buchrieser J. Considerable escape of SARS-CoV-2 Omicron to antibody neutralization. Nature (2022) 602(7898):671–5. doi: 10.1038/s41586-021-04389-z 35016199

[43] Cameroni E Bowen JE Rosen LE Saliba C Zepeda SK Culap K. Broadly neutralizing antibodies overcome SARS-CoV-2 Omicron antigenic shift. Nature (2022) 602(7898):664–70. doi: 10.1038/s41586-021-04386-2 PMC9531318 35016195

[44] Tarke A Coelho CH Zhang Z Dan JM Yu ED Methot N. SARS-CoV-2 vaccination induces immunological T cell memory able to cross-recognize variants from Alpha to Omicron. Cell (2022) 185(5):847–859 e11. doi: 10.1016/j.cell.2022.01.015 35139340 PMC8784649

[45] Reynolds CJ Pade C Gibbons JM Otter AD Lin KM Munoz Sandoval D. Immune boosting by B.1.1.529 (Omicron) depends on previous SARS-CoV-2 exposure. Science (2022) 377(6603):eabq1841. doi: 10.1126/science.abq1841 35699621 PMC9210451

[46] Tarke A Sidney J Kidd CK Dan JM Ramirez SI Yu ED. Comprehensive analysis of T cell immunodominance and immunoprevalence of SARS-CoV-2 epitopes in COVID-19 cases. Cell Rep Med (2021) 2(2):100204. doi: 10.1016/j.xcrm.2021.100204 33521695 PMC7837622

[47] Haney D Quigley MF Asher TE Ambrozak DR Gostick E Price DA. Isolation of viable antigen-specific CD8+ T cells based on membrane-bound tumor necrosis factor (TNF)-alpha expression. J Immunol Methods (2011) 369(1-2):33–41. doi: 10.1016/j.jim.2011.04.003 21501617 PMC3116017

[48] Migliori E Chang M Muranski P. Restoring antiviral immunity with adoptive transfer of *ex-vivo* generated T cells. Curr Opin Hematol (2018) 25(6):486–93. doi: 10.1097/MOH.0000000000000461 30281036

[49] Bollard CM Kuehnle I Leen A Rooney CM Heslop HE. Adoptive immunotherapy for posttransplantation viral infections. Biol Blood Marrow Transplant (2004) 10(3):143–55. doi: 10.1016/j.bbmt.2003.09.017 14993880

[50] de Candia P Prattichizzo F Garavelli S Matarese G. T cells: warriors of SARS-coV-2 infection. Trends Immunol (2021) 42(1):18–30. doi: 10.1016/j.it.2020.11.002 33277181 PMC7664351

[51] Liu R Wang Y Li J Han H Xia Z Liu F. Decreased T cell populations contribute to the increased severity of COVID-19. Clin Chim Acta (2020) 508:110–4. doi: 10.1016/j.cca.2020.05.019 PMC7219428 32405080

[52] Marcus N Frizinsky S Hagin D Ovadia A Hanna S Farkash M. Minor clinical impact of COVID-19 pandemic on patients with primary immunodeficiency in Israel. Front Immunol (2021) 11. doi: 10.3389/fimmu.2020.614086 PMC7840610 33519822

[53] Yazdanpanah F Hamblin MR Rezaei N. The immune system and COVID-19: Friend or foe? Life Sci (2020) 256:117900. doi: 10.1016/j.lfs.2020.117900 32502542 PMC7266583

[54] Dan JM Mateus J Kato Y Hastie KM Yu ED Faliti CE. Immunological memory to SARS-CoV-2 assessed for up to 8 months after infection. Science (2021) 371:587. doi: 10.1126/science.abf4063 PMC7919858 33408181

[55] Rucinski SL Binnicker MJ Thomas AS Patel R. Seasonality of Coronavirus 229E, HKU1, NL63, and OC43 From 2014 to 2020. Mayo Clin Proc (2020) 95(8):1701–3. doi: 10.1016/j.mayocp.2020.05.032 PMC7275147 32753142

[56] Moss P. The T cell immune response against SARS-CoV-2. Nat Immunol (2022) 23(2):186–93. doi: 10.1038/s41590-021-01122-w 35105982

[57] Rock KL Reits E Neefjes J. Present yourself! By MHC class I and MHC class II molecules. Trends Immunol (2016) 37(11):724–37. doi: 10.1016/j.it.2016.08.010 PMC5159193 27614798

[58] Nielsen M Lund O Buus S Lundegaard C. MHC class II epitope predictive algorithms. Immunology (2010) 130(3):319–28. doi: 10.1111/j.1365-2567.2010.03268.x PMC2913211 20408898

[59] Kuthuru O Baxter AE Herati RS Oldridge DA Gouma S Hicks P. Rapid induction of antigen-specific CD4 T cells is associated with coordinated humoral and cellular immunity to SARS-CoV-2 mRNA vaccination. Immunity (2021) 54:2133–42. doi: 10.1016/j.immuni.2021.08.001 PMC8361141 34453880

[60] Taus E Hofmann C Ibarrondo FJ Hausner MA Fulcher JA Krogstad P. Dominant CD8+ T cell nucleocapsid targeting in SARS-coV-2 infection and broad spike targeting from vaccination. Front Immunol (2022) 13:506. doi: 10.3389/fimmu.2022.835830 PMC8902813 35273611

[61] Peng Y Felce SL Dong D Penkava F Mentzer AJ Yao X. An immunodominant NP105–113-B*07:02 cytotoxic T cell response controls viral replication and is associated with less severe COVID-19 disease. Nat Immunol (2022) 23(1):50–61. doi: 10.1038/s41590-021-01084-z 34853448 PMC8709787

[62] Jing L Wu X Krist MP Hsiang TY Campbell VL McClurkan CL. T cell response to intact SARS-CoV-2 includes coronavirus cross-reactive and variant-specific components. JCI Insight (2022) 7(6). doi: 10.1172/jci.insight.158126 PMC8986086 35133988

[63] Woldemeskel BA Dykema AG Garliss CC Cherfils S Smith KN Blankson JN. CD4+ T cells from COVID-19 mRNA vaccine recipients recognize a conserved epitope present in diverse coronaviruses. J Clin Invest (2022) 132(5). doi: 10.1172/JCI156083 PMC8884904 35061630

[64] Keller MD Harris KM Jensen-Wachspress MA Kankate VV Lang H Lazarski CA. SARS-CoV-2–specific T cells are rapidly expanded for therapeutic use and target conserved regions of the membrane protein. Blood (2020) 136(25):2905–17. doi: 10.1182/blood.2020008488 PMC7746091 33331927

