# Supplementary material for: The prospect of universal coronavirus immunity: characterization of reciprocal and non-reciprocal T cell responses against SARS-CoV2 and common human coronaviruses
Source: Front Immunol. 2023 Oct 13;14:1212203. doi: 10.3389/fimmu.2023.1212203 (PMC10612330; doi:10.3389/fimmu.2023.1212203)
Supplement: Supplementary file 1 [file DataSheet_1.docx]

**
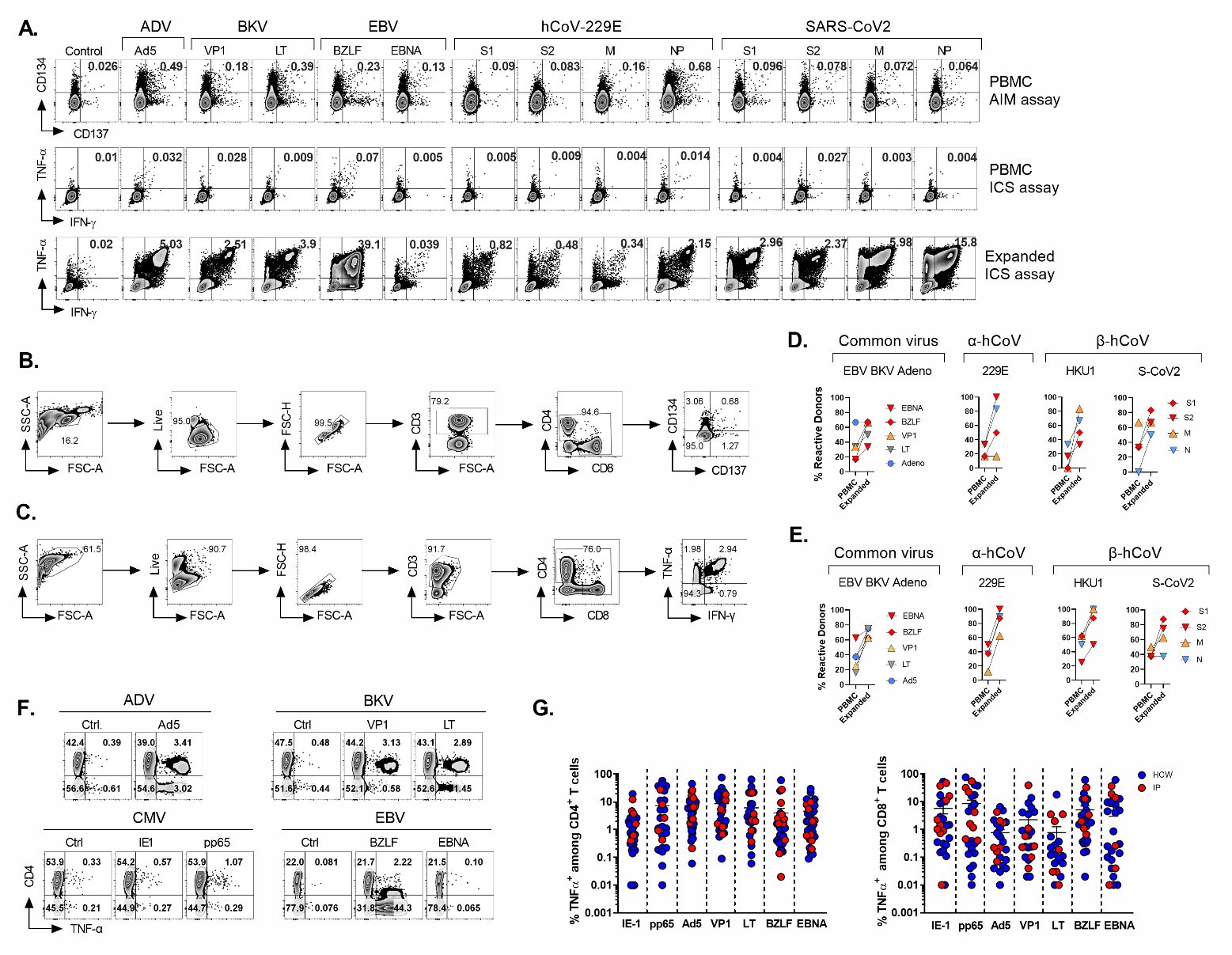
**

**Fig. S1: Ex vivo T cell responses as a measure of immunocompetence.** PBMCs from each donor (n=8) were stimulated with indicated antigen and frequency of antigen-specific T cells were measured between unexpanded (Day 0) and expanded (Day 14) T cells. **A**. Representative dot plot examples of AIM (day 0, upper row) and cytokine (day 0 and day 14 at middle and lower row, respectively) staining of reactive CD3^+^ T cells. **B-C.** Gating strategy to examine the frequency of antigen-specific T cells using AIM assay **(B)** or ICS assay **(C)**. **D.** Comparison of the sensitivity to measure immunocompetence between unexpanded and expanded T cells by ICS assay against indicated antigens (common viruses, α-hCoVs, β-hCoVs & SARS-CoV2)**. E.** Comparison of the sensitivity to measure immunocompetence between unexpanded and expanded T cells by AIM assay (CD134^+^CD137^+^CD4^+^ T cells) or ICS assay respectively. **F.** Reactivity of *ex vivo* expanded PBMCs from the same HCW collected pre-COVID-19 primed with peptide mixes derived from the immunodominant antigens of Adenovirus (Ad5), BK polyomavirus (VP1 and LT), CMV (IE1 and pp65) and EBV (BZLF1 and EBNA1) gated identically as in Figure 1. **G.** Frequencies of virus reactive CD4^+^ T cells (Left panel) and CD8^+^ T cells (Right panel) against indicated antigens in HCW and IP. Blue dots represent HCW, red dots represent IP. Reactive donors were defined as follows: For Day 0 Cytokine release assay and AIM assay, donors displaying more than 0.01% TNF^+^ T cells than the background were considered reactive; while for expanded T cell cytokine assay, donors displaying more than 0.01% TNFα^+^ T cells than the background were considered reactive.


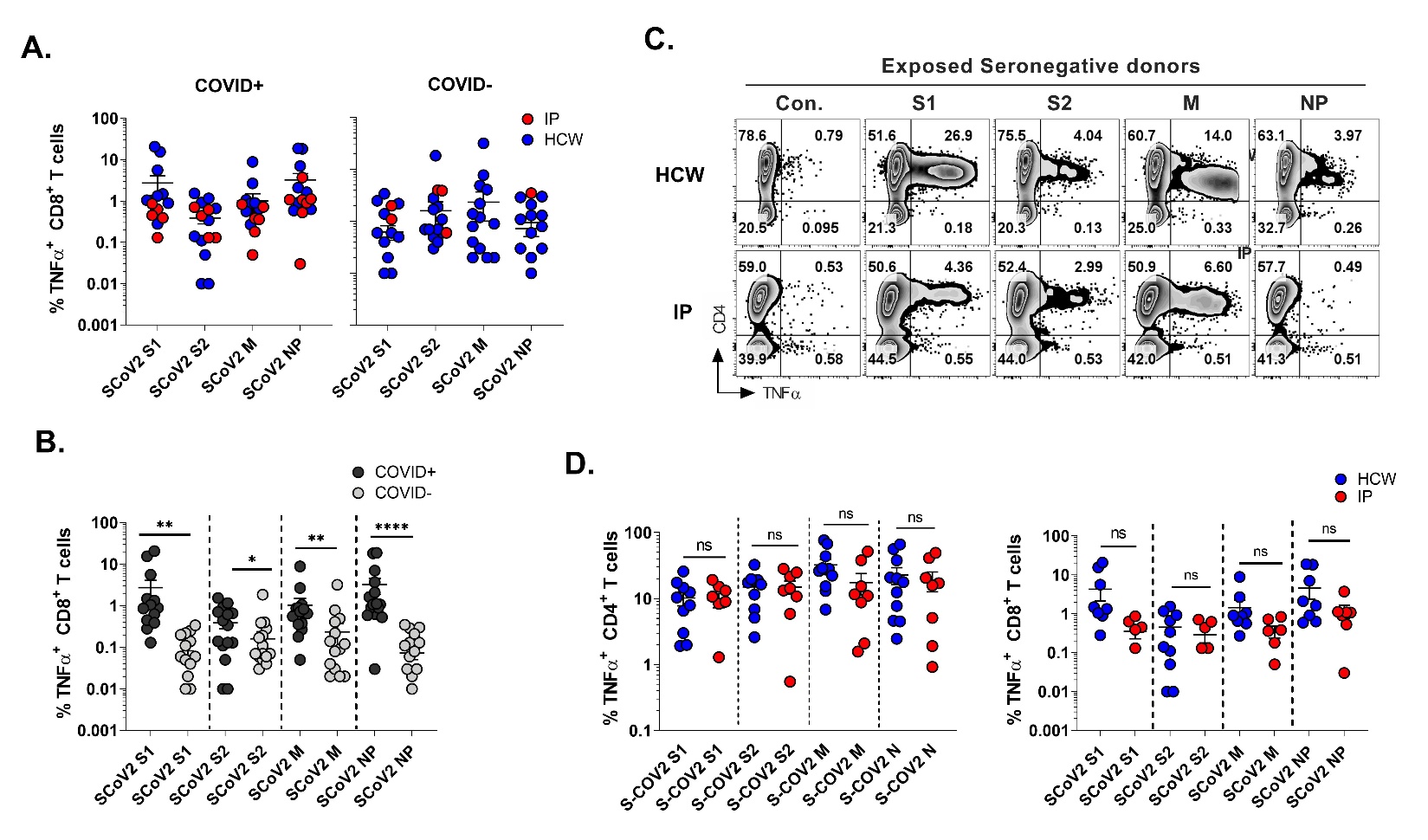


**Fig. S2: T cell responses against SARS-CoV-2 antigens.** **A.**Frequencies of SARS-CoV-2 reactive CD8^+^ T cells against Spike 1, Spike 2, Membrane and Nucleocapsid proteins in COVID+ donors. (Left panel) and COVID- donors (Right panel). Blue dots are HCW, red dots are IP. **B.** Comparison of frequencies of SARS-CoV-2 reactive CD8+ T cells against indicated proteins between COVID+ and COVID- donors. **C.**Recognition of indicated SARS-CoV-2 peptide mixes in cultures from a PCR negative and seronegative healthcare worker (#1018) with highly probable exposure history (upper panel) and a seronegative immunocompromised patient (#1032) mounting robust T cells response against SARS-CoV-2 (lower panel). **D.**Comparison of frequencies of SARS-CoV-2 reactive CD4^+^ and CD8^+^ T cells against indicated proteins between HCW and IP.  Statistically significant differences of reactivity were determined by Mann-Whitney test. **P < 0.01, ***P < 0.001.

**
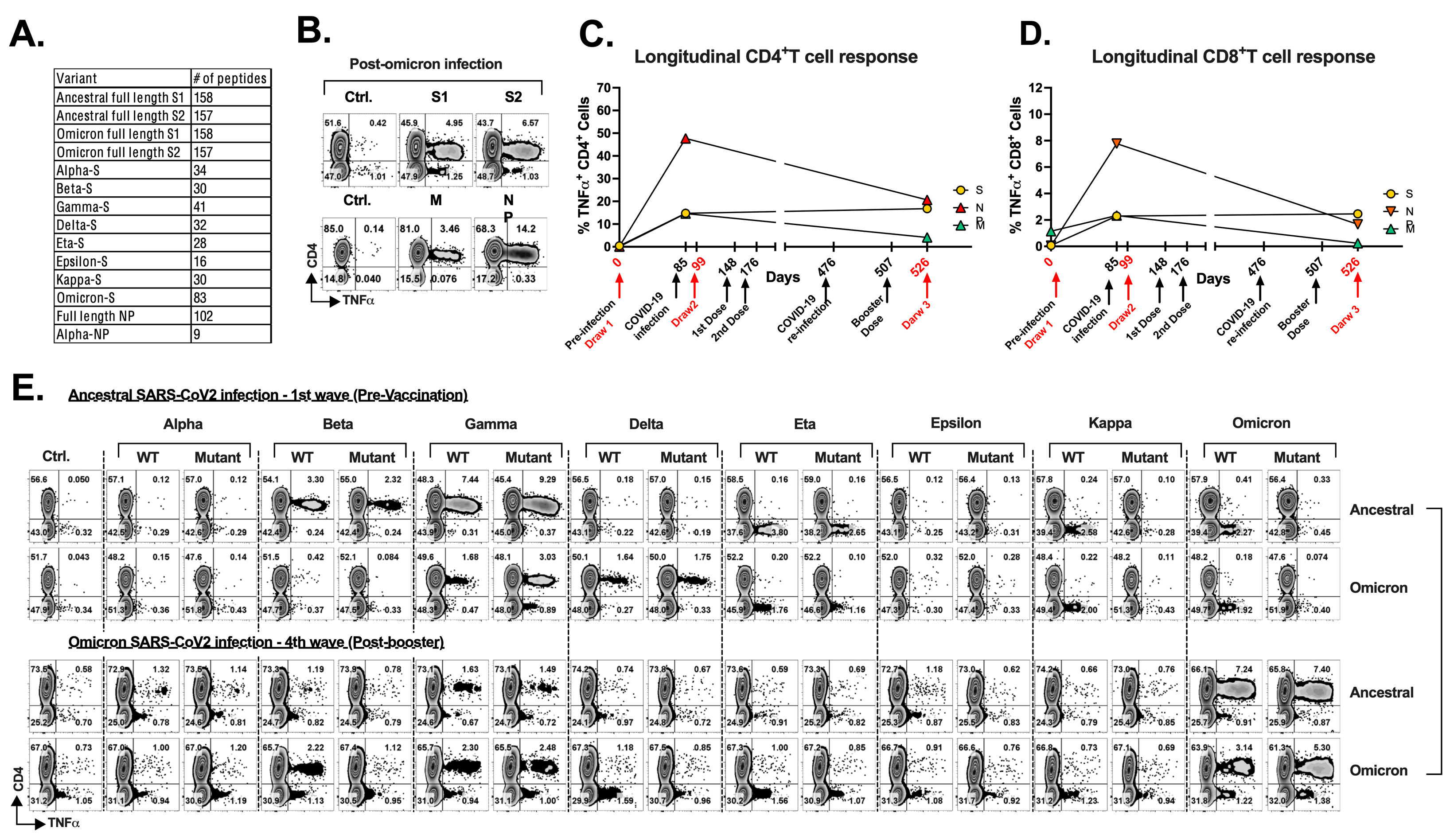
**

**Fig. S3: Comparison of T cell response to variants post ancestral and omicron infection in a healthcare worker.**  **A.** Table showing number of peptides in pepmixes of indicated antigens. **B.** Zebra plots showing T cell response against Membrane and Nucleocapsid proteins after omicron infection. **C-D**. Longitudinal analysis of CD4^+^ T cell **(C.)** and CD8^+^ T cell **(D.)** response analysis of donor 1008. **E.** Zebra plots depicting T cell response to SARS CoV2 variants. Top panel represents response after infection during 1^st^ wave before vaccination, whereas response after infection during fourth wave is shown in bottom panel.


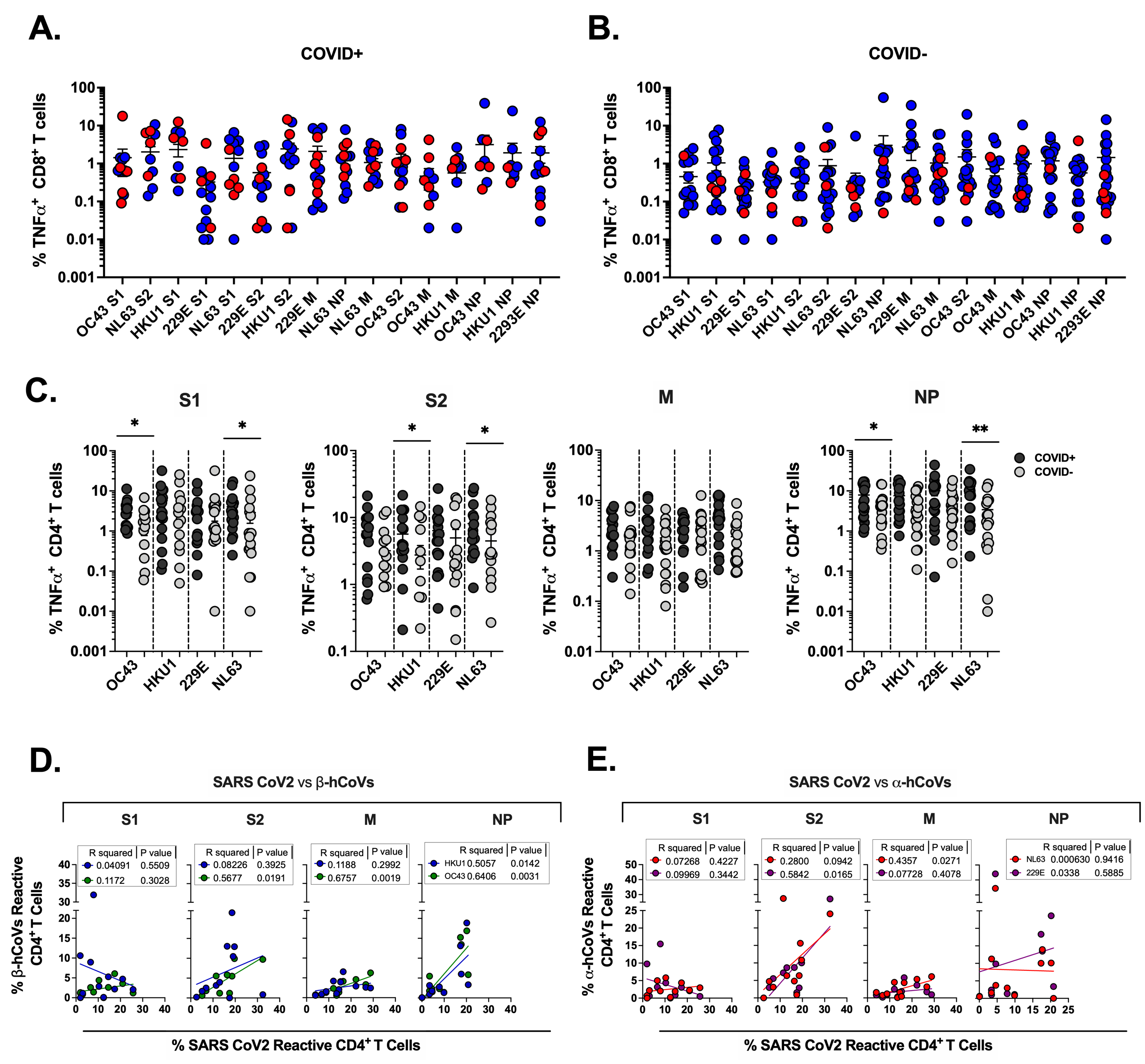


**Fig. S4: CD8+ T cell responses against SARS-CoV2 antigens.** **A.** Frequencies of reactive CD8^+^ T cells against S1, S2, M, and NP proteins from human coronavirus (hCoVS) in COVID+ donors. **B.** Frequencies of reactive CD8^+^ T cells against S1, S2, M, and NP proteins from human coronavirus (hCoVS) in COVID- donors. **C.** Comparison of reactivity between COVID+ and COVID- donors against indicated antigens. Statistically significant differences of reactivity were determined by Mann-Whitney test. Horizontal lines indicate the mean ± SEM. *P < 0.05, **P < 0.01, ***P < 0.001. **D.** Linear correlation between SARS-CoV2 and α-coronavirus reactivity in COVID+ healthcare workers. **E.** Linear correlation between SARS-CoV2 and α-coronavirus reactivity in COVID- healthcare workers.

**
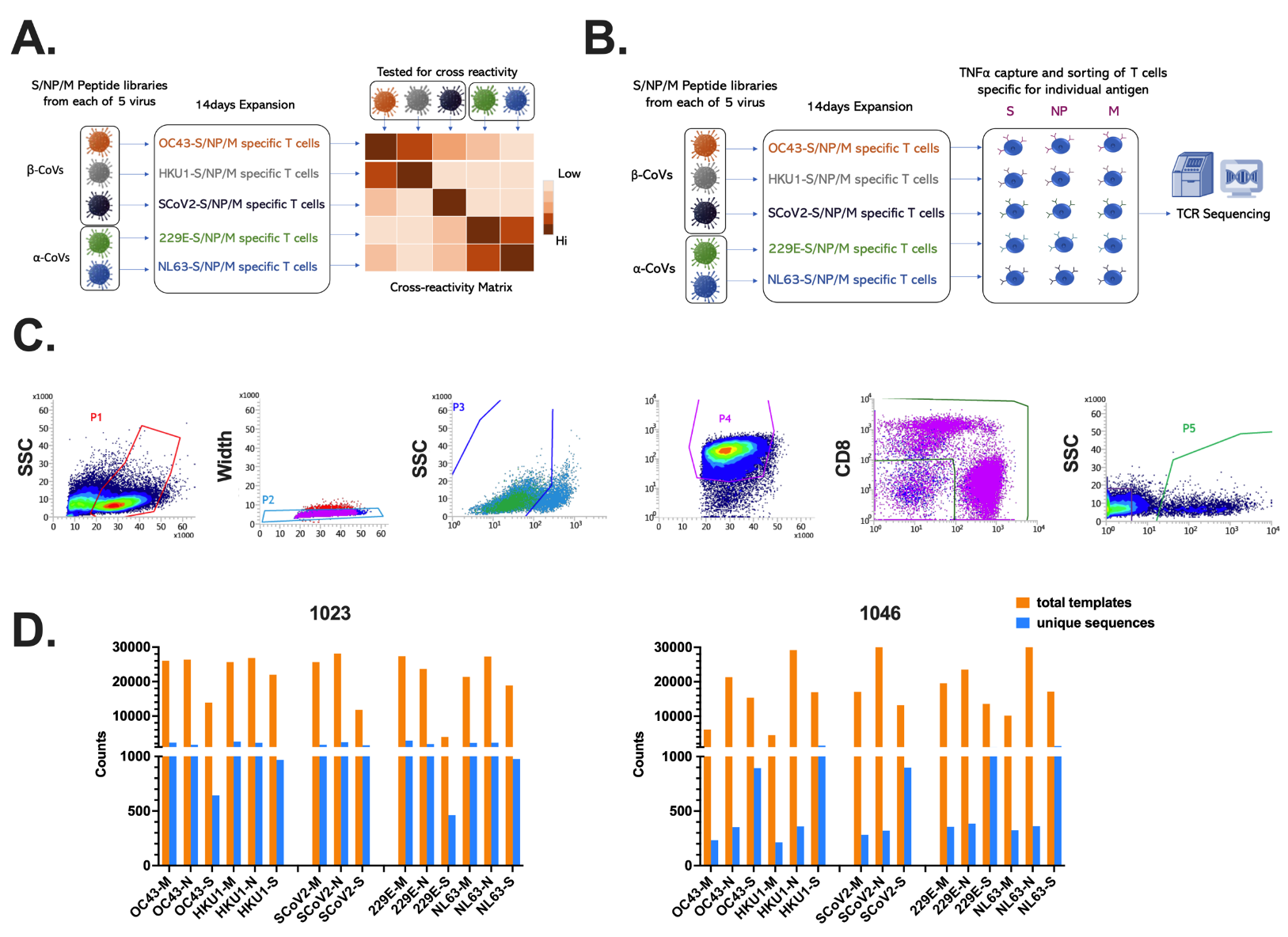
**

**Fig. S5: Experimental design for analyzing cross-reactivity and TCR repertoire. A.** Schematic diagram depicting experimental design to examine cross-reactivity among SARS-CoV2 and human coronaviruses. **B.** Schematic diagram showing strategy for TCR sequencing of antigen-specific T cells**. C.** Gating strategy for sorting virus specific T cells. **D.** Total template counts, and unique sequence counts for TCRs detected in all cultured samples from subject #1023 (Left) and #1046 (Right) against all three antigens (M, N, S) in all 5 CoVs.

**
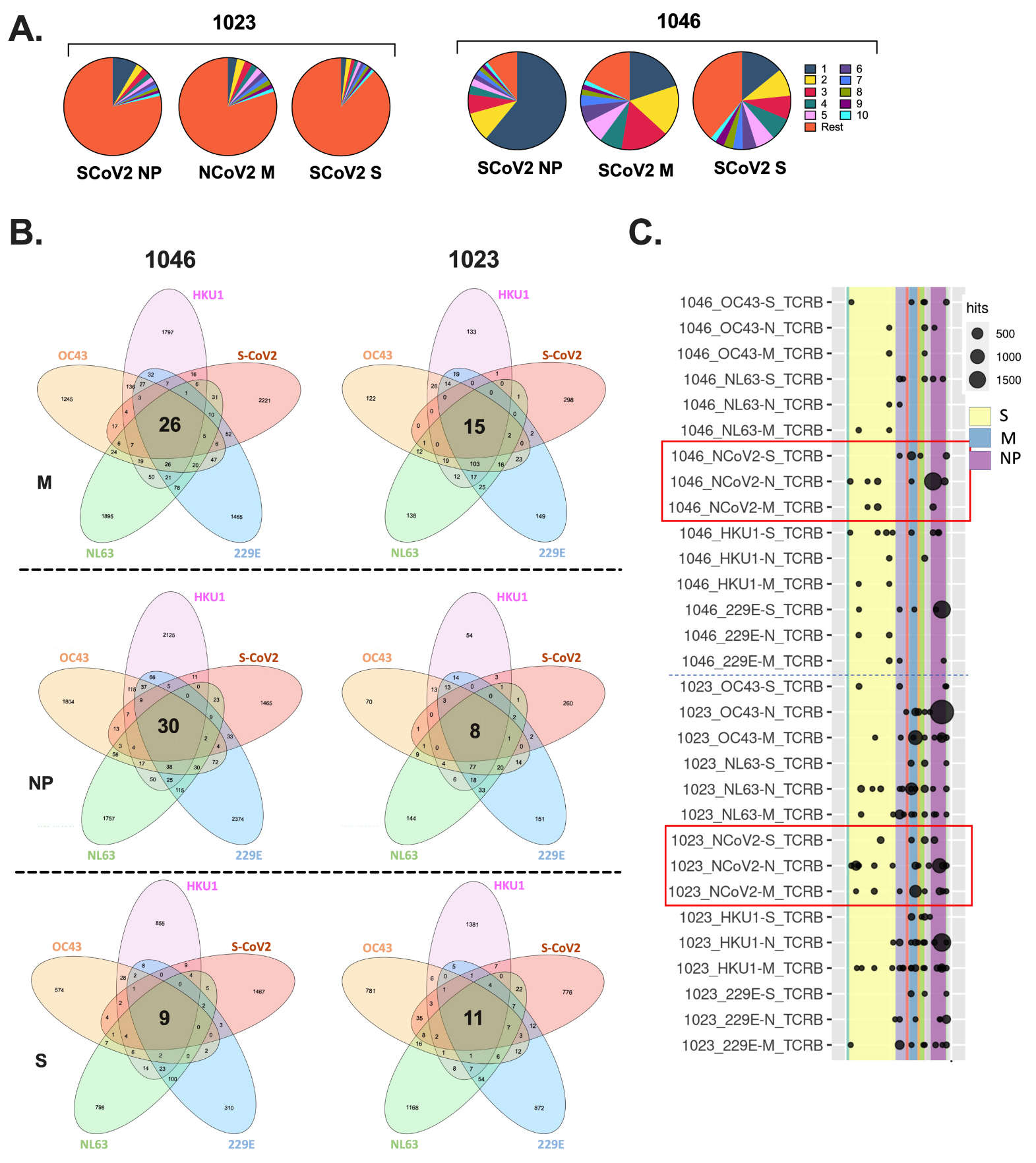
**

**Fig. S6: TCR repertoire analysis. A.** Pie charts showing proportion of top 10 dominant clones within antigen-specific TCR repertoire for both subjects. 1023 (Left) and 1046 (Right). **B.** Venn diagram showing number of TCRs shared between different coronaviruses. **C.** Mapping of Coronavirus specific TCRs in Adaptive Biotechnologies’ ImmuneCODE database containing SARS-CoV-2 specific TCRs identifies shared SARS-CoV2 TCRs along with potential cross-reactive TCRs.
